# Statistical Divergence and Paths Thereof to Socioeconomic Inequality and to Renewal Processes

**DOI:** 10.3390/e26070565

**Published:** 2024-06-30

**Authors:** Iddo Eliazar

**Affiliations:** School of Chemistry, Tel Aviv University, Tel Aviv 6997801, Israel; eliazar@tauex.tau.ac.il

**Keywords:** Kullback–Leibler divergence (relative entropy), Renyi divergence, f-divergence, Lorenz curve, inequality indices, Gini index, renewal processes, 02.50.-r (probability theory, stochastic processes, and statistics), 05.40.-a (fluctuation phenomena, random processes, noise, and Brownian motion), 89.65.-s (social and economic systems)

## Abstract

This paper establishes a general framework for measuring statistical divergence. Namely, with regard to a pair of random variables that share a common range of values: quantifying the distance of the statistical distribution of one random variable from that of the other. The general framework is then applied to the topics of socioeconomic inequality and renewal processes. The general framework and its applications are shown to yield and to relate to the following: f-divergence, Hellinger divergence, Renyi divergence, and Kullback–Leibler divergence (also known as relative entropy); the Lorenz curve and socioeconomic inequality indices; the Gini index and its generalizations; the divergence of renewal processes from the Poisson process; and the divergence of anomalous relaxation from regular relaxation. Presenting a ‘fresh’ perspective on statistical divergence, this paper offers its readers a simple and transparent construction of statistical-divergence gauges, as well as novel paths that lead from statistical divergence to the aforementioned topics.

## 1. Introduction

Measuring distances is of foundational importance in all fields of science and engineering. Arguably, measuring distances emerged with regard to points in planar geometry. Elevating from points in the plane to points in general spaces—e.g., Hilbert, Banach, and metric spaces—facilitates measuring distances between very general objects.

Even in the basic case of planar geometry, there are various ways of measuring distances. To illustrate this, envisage Manhattan (above 14th street). From an aerial perspective, the distance between two addresses in Manhattan is the Euclidean distance. From a pedestrian perspective, the distance between two addresses in Manhattan is the grid distance—which is attained by walking along avenues and streets (as pedestrians cannot walk through buildings).

Measuring distances is not at all confined to geometry—be it in the plane, or in general spaces. Indeed, consider a human society of interest and the distribution of wealth among its members. In a purely egalitarian society, the distribution of wealth is perfectly equal: all the members have exactly the same wealth. Of course, any real human society is not purely egalitarian. A key topic in economics and in the social sciences is quantifying *socioeconomic inequality* [1,2,3]. Namely, measuring the distance of the human society of interest from the ‘benchmark state’ of perfect equality. This topic is addressed by important notions including the *Lorenz curve* [4,5,6,7] and socioeconomic *inequality indices* [1,2,3,8,9].

Shifting from socioeconomics to statistics, and from distributions of wealth to statistical distributions: consider the statistical distributions of random variables that take values in a common range (e.g., the positive half-line). In this context, a key topic is measuring *statistical divergence* [10,11,12]: the distance of a statistical distribution of interest from a ‘benchmark’ statistical distribution. Mainly, this topic is addressed by the *Kullback–Leibler divergence* (also known as “relative entropy”) [13,14,15,16]; the *Renyi divergence* [17,18,19,20]; and the more general “*f-divergence*” [17,21,22,23,24,25,26]. Recent examples of the wide use of these divergences include [27,28,29,30,31,32,33,34,35,36,37,38,39,40,41,42].

This paper establishes a general framework for measuring statistical divergence. The general framework is presented in Section 2, and it has a high ‘return on investment’. Indeed, on the one hand, the framework is based on a simple and transparent construction. And, on the other hand, the framework yields potent gauges of statistical divergence. In particular, the framework leads to the aforementioned divergences. With the general framework established, the paper presents two applications of it.

Section 3 applies the general framework to the measurement of socioeconomic inequality. In particular, this application will show how the framework yields the aforementioned notions of the Lorenz curve and socioeconomic inequality indices; as well as the *Gini index*—perhaps the best known and the most widely applied socioeconomic inequality index [43,44,45,46,47,48]—and its generalizations.

Section 4 applies the general framework to *renewal processes* [49,50,51]. In particular, this application will show how the framework facilitates measuring the divergence of renewal processes from the *Poisson process* [52,53,54], and measuring the divergence of *anomalous relaxation* [55,56,57,58] from ‘regular’ exponential relaxation. In addition, this application will yield further relations to socioeconomic inequality indices, as well as further generalizations of the Gini index.

Each of the Section 2, Section 3 and Section 4 ends with a short summary. For a quick read of the paper, readers can go over these summaries.

The novelty of this paper is twofold. Firstly, the paper offers its readers a direct and transparently constructed path to statistical divergence. Thereof, the paper offers its readers further paths to socioeconomic inequality, to renewal processes, and to anomalous relaxation. The paper illuminates profound linkages, which are not straightforwardly apparent, between the different (and seemingly unrelated) topics it addresses.

The paper is written in a self-contained fashion, and its pre-requisites are basic calculus and basic probability. Thus, the paper is suitable for a wide range of audiences: theoreticians and practitioners alike, from diverse fields of science and engineering.

*A note about notation*. Throughout this paper, IID is an acronym for “independent and identically distributed” (random variables), and E[·] denotes expectation (namely, E[Z] is the mean of a given random variable *Z*).

## 2. General Framework

Consider a random variable *X* that takes values in a real range $R=(r_{low},r_{up})$, where $r_{low}$ is the range’s lower bound, $r_{up}$ is the range’s upper bound, and $-\infty \leq r_{low}<r_{up}\leq \infty$. Further consider a random variable *Y* that is independent of *X* and that takes values in the range $\left[r_{low},r_{up}\right]$. Namely, in addition to values in the range R, the random variable *Y* can also attain the lower-bound value $r_{low}$ and the upper-bound value $r_{up}$.

The goal of this paper is to measure the statistical divergence of the random variable *Y* from the random variable *X*. In other words, the goal is to quantify the extent by which the statistical distribution of the random variable *Y* deviates from the statistical distribution of the random variable *X*.

Henceforth, the distribution functions of *X* and *Y* are denoted, respectively, by $A\left(r\right)=\mathrm{Pr}\left(X\leq r\right)$ (r∈R) and by $B\left(r\right)=\mathrm{Pr}\left(Y\leq r\right)$ (r∈R). The distribution function $A\left(r\right)$ is assumed to be increasing, and hence it has an increasing inverse function $A^{-1}\left(u\right)$ (0<u<1). In addition, the probability that the random variables *X* and *Y* coincide is assumed to be zero, $\mathrm{Pr}\left(X=Y\right)=0$; this assumption holds whenever the distribution function $A\left(r\right)$ is continuous.

We shall measure the statistical divergence of *Y* from *X* via the curve

(1) $$C\left(u\right)=B\left[A^{-1}\left(u\right)\right]$$

(0<u<1). It is straightforward to observe that the curve $C\left(u\right)$ is non-decreasing, and that its boundary values are $\lim_{u\to 0}C\left(u\right)=0$ and $\lim_{u\to 1}C\left(u\right)=1$. These observations imply that the curve $C\left(u\right)$ manifests a distribution function over the unit interval. Specifically, $C\left(u\right)=\mathrm{Pr}\left(U\leq u\right)$, where *U* is a random variable that takes values in the unit interval $\left[0,1\right]$.

In the case that the distribution functions of *X* and *Y* are smooth, their corresponding density functions are denoted, respectively, by $a\left(r\right)=A^{\prime }\left(r\right)$ (r∈R) and by $b\left(r\right)=B^{\prime }\left(r\right)$ (r∈R). In turn, differentiating Equation (1) yields

(2) $$C^{\prime }\left(u\right)=\frac{b\left[A^{-1}\left(u\right)\right]}{a\left[A^{-1}\left(u\right)\right]}$$

(0<u<1). Namely, $C^{\prime }\left(u\right)$ is the density function of the random variable *U*. (As the distribution function $A\left(r\right)$ is assumed to be increasing, the density $a\left(r\right)$ is positive, and hence: the denominator appearing on the right-hand side of Equation (2) is positive, and the ratio is well-defined).

Statistical distributions that are defined over the unit interval have a natural ‘benchmark’: the uniform distribution—the unique statistical distribution that assigns all possible outcomes the same likelihood of occurrence. The uniform distribution is characterized by the linear distribution function $C_{*}\left(u\right)=u$ (0<u<1), as well as by the flat density function $C_{*}^{\prime }\left(u\right)=1$ (0<u<1). In what follows, we set $U_{*}$ to be a random variable that is uniformly distributed over the unit interval, $\mathrm{Pr}\left(U_{*}\leq u\right)=u$.

Equation (1) implies that the random variables *X* and *Y* are equal in law, $A\left(r\right)\equiv B\left(r\right)$, if and only if the random variables *U* and $U_{*}$ are equal in law, $C\left(u\right)\equiv u$. Consequently, the statistical divergence of *Y* from *X* can be measured ‘by proxy’ as follows: quantifying the deviation of the statistical distribution of the random variable *U* from the uniform distribution. We shall do so using three methods: area—Section 2.1; moments—Section 2.2; and coincidence—Section 2.4.

### 2.1. Area

The graphs of distribution functions that are defined over the unit interval reside in the unit square, where they ‘climb’ from the square’s bottom-left corner to its top-right corner. Namely, the 2D coordinates of the unit-square’s bottom-left corner are $\left(0,0\right)$, and the 2D coordinates of the top-right corner are $\left(1,1\right)$. The distribution function of the uniform distribution, $C_{*}\left(u\right)=u$ (0<u<1), is the diagonal line of the unit square. This line splits the unit square into two triangles: the triangle below the diagonal line, whose area is $\frac{1}{2}$; and the triangle above the diagonal line, whose area is also $\frac{1}{2}$. Thus, the difference between the area of the upper triangle and the area of the lower triangle is zero.

Similarly to the diagonal line, the curve $C\left(u\right)$ splits the unit square into two sets: the ‘lower set’, comprising the square’s points that are below the curve; and the ‘upper set’, comprising the square’s points that are above the curve. A derivation detailed in Appendix A asserts that the area of the lower set is $\int_{0}^{1}C\left(u\right)du=\mathrm{Pr}\left(Y\leq X\right)$. In turn, as the square’s total area is one, the area of the upper set is $1-\mathrm{Pr}\left(Y\leq X\right)=\mathrm{Pr}\left(Y>X\right)$. Thus, the difference between the area of the upper set and the area of the lower set is the quantity

(3) $$\Delta \left(Y||X\right)=\mathrm{Pr}\left(Y>X\right)-\mathrm{Pr}\left(Y\leq X\right).$$

With regard to Equation (3), recall that the random variables *X* and *Y* are considered to be mutually independent. In terms of the curve $C\left(u\right)$, the quantity of Equation (3) admits the formulation $\Delta \left(Y||X\right)=1-2\int_{0}^{1}C\left(u\right)du$.

The quantity $\Delta \left(Y||X\right)$ takes values in the range [−1,1]. The quantity $\Delta \left(Y||X\right)$ attains its lower bound if and only if the random variable *Y* equals its lower bound with probability one: $\Delta \left(Y||X\right)=-1\Leftrightarrow \mathrm{Pr}\left(Y=r_{low}\right)=1$. Antithetically, the quantity $\Delta \left(Y||X\right)$ attains its upper bound if and only if the random variable *Y* equals its upper bound with probability one: $\Delta \left(Y||X\right)=1\Leftrightarrow \mathrm{Pr}\left(Y=r_{up}\right)=1$. The quantity $\Delta \left(Y||X\right)$ is zero if—but not only if—the random variables *X* and *Y* are equal in law: $A\left(r\right)\equiv B\left(r\right)\Rightarrow \Delta \left(Y||X\right)=0$ (this implication uses the aforementioned assumption $\mathrm{Pr}\left(Y=X\right)=0$).

We illustrate the fact that a zero quantity $\Delta \left(Y||X\right)$ does not imply that the random variables *X* and *Y* are equal in law. To that end, consider the random variables *X* and *Y* to be symmetric: X=−X and Y=−Y, the equalities being in law. The symmetry of the random variables *X* and *Y* implies that $\mathrm{Pr}\left(Y>X\right)=\mathrm{Pr}\left(Y\leq X\right)$ (this implication also uses the aforementioned assumption $\mathrm{Pr}\left(Y=X\right)=0$), and hence $\Delta \left(Y||X\right)=0$. However, the symmetry of *X* and *Y* does not imply that these random variables are equal in law.

The quantity $\Delta \left(Y||X\right)$ is a ‘first-order measurement’ of the statistical divergence of the random variable *Y* from the random variable *X*. Indeed, the quantity $\Delta \left(Y||X\right)$ does not characterize the case of a zero statistical divergence, i.e., the case where the random variables *X* and *Y* are equal in law. In the next subsection, we shall elevate from the ‘first-order measurement’ $\Delta \left(Y||X\right)$ to ‘higher-order measurements’.

### 2.2. Moments

Consider a random variable ξ that takes values in the unit interval, and whose statistical distribution is governed by the density function $\phi \left(u\right)$ (0<u<1). The moments of the random variable ξ are the sequence $E\left[\xi^{m}\right]=\int_{0}^{1}u^{m}\phi \left(u\right)du$ (m=1,2,3,⋯). The Hausdorff moment problem asserts that the moments of the random variable ξ characterize its statistical distribution [59]. Namely, if another random variable—that also takes values in the unit interval—has the same moments as ξ, then the two random variables are equal in law.

In this subsection we shall apply the Hausdorff moment problem in order to measure the statistical divergence of the random variable *Y* from the random variable *X*. In what follows, $\left\{X_{1},\cdots ,X_{m}\right\}$ denote *m* IID copies of the random variable *X*; these IID copies are considered to be independent of the random variable *Y*.

As above, $U_{*}$ denotes a random variable that is uniformly distributed over the unit interval. As the density function of the uniform distribution is $C_{*}^{\prime }\left(u\right)=1$, the moments of the random variable $U_{*}$ are $E\left[U_{*}^{m}\right]=\frac{1}{m+1}$. A derivation detailed in Appendix A asserts that the moments of the random variable *U* are $E\left[U^{m}\right]=\mathrm{Pr}\left(X_{1},\cdots ,X_{m}\leq Y\right)$; namely, the moment $E[U^{m}]$ is the probability that the *m* IID copies of *X* are all no larger than *Y*. Consequently, multiplying the difference of the moments $E\left[U^{m}\right]-E\left[U_{*}^{m}\right]$ by the factor m+1 yields the quantity

(4) $$\alpha_{m}\left(Y||X\right)=\left(m+1\right)\mathrm{Pr}\left(X_{1},\cdots ,X_{m}\leq Y\right)-1.$$

The quantity $\alpha_{m}\left(Y||X\right)$ involves the probability $p_{m}=\mathrm{Pr}\left(X_{1},\cdots ,X_{m}\leq Y\right)$. Being a probability, $p_{m}$ takes values in the unit interval $\left[0,1\right]$, and hence the quantity $\alpha_{m}\left(Y||X\right)$ takes values in the range [−1,m]. Moreover, the quantity $\alpha_{m}\left(Y||X\right)$ attains its lower bound −1 if and only if $p_{m}=0$, i.e., if and only if the random variable *Y* equals its lower bound $r_{low}$ with probability one: $\alpha_{m}\left(Y||X\right)=-1\Leftrightarrow \mathrm{Pr}\left(Y=r_{low}\right)=1$. And, antithetically, the quantity $\alpha_{m}\left(Y||X\right)$ attains its upper bound *m* if and only if $p_{m}=1$, i.e., if and only if the random variable *Y* equals its upper bound $r_{up}$ with probability one: $\alpha_{m}\left(Y||X\right)=m\Leftrightarrow \mathrm{Pr}\left(Y=r_{up}\right)=1$.

As the random variables $U_{*}$ and *U* take values in the unit interval, so do the random variables $\;1-U_{*}$ and 1−U. The random variable $\;1-U_{*}$ is uniformly distributed, and hence its moments are $E\left[{\left(1-U\right)}_{*}^{m}\right]=\frac{1}{m+1}$. A derivation detailed in Appendix A asserts that the moments of the random variable 1−U are $E\left[{\left(1-U\right)}^{m}\right]=\mathrm{Pr}\left(X_{1},\cdots ,X_{m}>Y\right)$; namely, the moment $E[{\left(1-U\right)}^{m}]$ is the probability that the *m* IID copies of *X* are all larger than *Y*. Consequently, multiplying the difference of moments $E\left[{\left(1-U_{*}\right)}^{m}\right]-E\left[{\left(1-U\right)}^{m}\right]$ by the factor m+1 yields the quantity

(5) $$\beta_{m}\left(Y||X\right)=1-\left(m+1\right)\mathrm{Pr}\left(X_{1},\cdots ,X_{m}>Y\right).$$

The quantity $\beta_{m}\left(Y||X\right)$ involves the probability $q_{m}=\mathrm{Pr}\left(X_{1},\cdots ,X_{m}>Y\right)$. Being a probability, $q_{m}$ takes values in the unit interval $\left[0,1\right]$, and hence the quantity $\beta_{m}\left(Y||X\right)$ takes values in the range [−m,1]. Moreover, the quantity $\beta_{m}\left(Y||X\right)$ attains its lower bound −m if and only if $q_{m}=1$, i.e., if and only if the random variable *Y* equals its lower bound $r_{low}$ with probability one: $\beta_{m}\left(Y||X\right)=-m\Leftrightarrow \mathrm{Pr}\left(Y=r_{low}\right)=1$. And, antithetically, the quantity $\beta_{m}\left(Y||X\right)$ attains its upper bound 1 if and only if $q_{m}=0$, i.e., if and only if the random variable *Y* equals its upper bound $r_{up}$ with probability one: $\beta_{m}\left(Y||X\right)=1\Leftrightarrow \mathrm{Pr}\left(Y=r_{up}\right)=1$.

The Hausdorff moment problem implies that the random variables *U* and $U_{*}$ are equal in law if and only if $E\left[U^{m}\right]=E\left[U_{*}^{m}\right]$ (m=1,2,3,⋯), as well as if and only if $E\left[{\left(1-U\right)}^{m}\right]=E\left[{\left(1-U_{*}\right)}^{m}\right]$ (m=1,2,3,⋯). In turn, we obtain that the random variables *X* and *Y* are equal in law if and only if the quantities $\alpha_{m}\left(Y||X\right)$ are all zero, as well as if and only if the quantities $\beta_{m}\left(Y||X\right)$ are all zero: $A\left(r\right)\equiv B\left(r\right)\Leftrightarrow \alpha_{m}\left(Y||X\right)=0$ (m=1,2,3,⋯); and $A\left(r\right)\equiv B\left(r\right)\Leftrightarrow \beta_{m}\left(Y||X\right)=0$ (m=1,2,3,⋯). Thus, collectively, the quantities $\alpha_{m}\left(Y||X\right)$, as well as the quantities $\beta_{m}\left(Y||X\right)$, characterize the case of a zero statistical divergence of the random variable *Y* from the random variable *X*.

For m=1, Equations (4) and (5) imply that

(6) $$\alpha_{1}\left(Y||X\right)=\beta_{1}\left(Y||X\right)=\Delta \left(Y||X\right),$$

where $\Delta \left(Y||X\right)$ is the quantity of Equation (3) (this implication uses the aforementioned assumption $\mathrm{Pr}\left(Y=X\right)=0$). As noted in Section 2.1, the quantity $\Delta \left(Y||X\right)$ is a ‘first-order measurement’ of the statistical divergence of the random variable *Y* from the random variable *X*. The quantities $\alpha_{m}\left(Y||X\right)$ and $\beta_{m}\left(Y||X\right)$ are ‘higher-order measurements’ of this statistical divergence.

To conclude this subsection, we underscore the fact that no use of the moments of the random variables *X* and *Y* was made here. Indeed, in general, the random variables *X* and *Y* may or may not have well-defined moments. This subsection used the (well-defined) moments of the random variables *U* and 1−U in order to ‘harvest information’ regarding the statistical divergence of the random variable *Y* from the random variable *X*. The information that was harvested appeared in terms of the probabilities $\mathrm{Pr}\left(X_{1},\cdots ,X_{m}\leq Y\right)$ and $\mathrm{Pr}\left(X_{1},\cdots ,X_{m}>Y\right)$ (rather than in terms of moments of the random variables *X* and *Y*).

### 2.3. Weighted-Average Representations

The previous subsection established the quantities $\alpha_{m}\left(Y||X\right)$ (m=1,2,3,⋯) and the quantities $\beta_{m}\left(Y||X\right)$ (m=1,2,3,⋯). In this subsection, we further establish weighted-average representations of these quantities. As in the previous subsection, $\left\{X_{1},\cdots ,X_{m}\right\}$ denote *m* IID copies of the random variable *X* that are independent of the random variable *Y*.

The quantity $\alpha_{m}\left(Y||X\right)$ of Equation (4) involves the maximal random variable $\max\left\{X_{1},\cdots ,X_{m}\right\}$. Indeed, the probability appearing in the right-hand side of Equation (4) is $\mathrm{Pr}\left(\max\left\{X_{1},\cdots ,X_{m}\right\}\leq Y\right)$. Set ${\overset{ˇ}{a}}_{m}\left(r\right)$ (r∈R) to denote the density function of the maximal random variable $\max\left\{X_{1},\cdots ,X_{m}\right\}$. Then, using this density function, a derivation detailed in Appendix A asserts that

(7) $$\alpha_{m}\left(Y||X\right)=\int_{R}\left[A\left(r\right)-B\left(r\right)\right]{\overset{ˇ}{a}}_{m}\left(r\right)dr.$$

The quantity $\beta_{m}\left(Y||X\right)$ in Equation (5) involves the minimal random variable $\min\left\{X_{1},\cdots ,X_{m}\right\}$. Indeed, the probability appearing in the right-hand side of Equation (5) is $\mathrm{Pr}\left(\min\left\{X_{1},\cdots ,X_{m}\right\}>Y\right)$. Set ${\hat{a}}_{m}\left(r\right)$ (r∈R) to denote the density function of the minimal random variable $\min\left\{X_{1},\cdots ,X_{m}\right\}$. Then, using this density function, a derivation detailed in Appendix A asserts that

(8) $$\beta_{m}\left(Y||X\right)=\int_{R}\left[A\left(r\right)-B\left(r\right)\right]{\hat{a}}_{m}\left(r\right)dr.$$

For m=1, the maximal random variable $\max\left\{X_{1},\cdots ,X_{m}\right\}$ and the minimal random variable $\min\left\{X_{1},\cdots ,X_{m}\right\}$ are both equal, in law, to the random variable *X*. Consequently, ${\overset{ˇ}{a}}_{m}\left(r\right)={\hat{a}}_{m}\left(r\right)=a\left(r\right)$, and hence Equations (7), (8) and Equation (6) imply that

(9) $$\Delta \left(Y||X\right)=\int_{R}\left[A\left(r\right)-B\left(r\right)\right]a\left(r\right)dr.$$

Equations (7)–(9) manifest weighted-average representations for the quantities appearing in them. In these representations, the averages are of the difference between the distribution functions of the random variables *X* and *Y*. Moreover, the averaging weights are as follows: the density function of the maximal random variable $\max\left\{X_{1},\cdots ,X_{m}\right\}$ in Equation (7); the density function of the minimal random variable $\min\left\{X_{1},\cdots ,X_{m}\right\}$ in Equation (8); and the density function of the random variable *X* in Equation (9).

### 2.4. Coincidence

As above, consider a random variable ξ that takes values in the unit interval, and whose statistical distribution is governed by the density function $\phi \left(u\right)$ (0<u<1). Further consider *m* IID simulations of the random variable ξ (m=2,3,4,⋯). The likelihood that the *m* simulations will all yield the same outcome is $L_{m}\left[\xi \right]=\int_{0}^{1}\phi {\left(u\right)}^{m}du$. We term $L_{m}\left[\xi \right]$ the “coincidence likelihood”, of order *m*, of the random variable ξ. In this subsection we shall apply coincidence likelihoods in order to measure the statistical divergence of the random variable *Y* from the random variable *X*.

As above, $U_{*}$ denotes a random variable that is uniformly distributed over the unit interval. As the density function of the uniform distribution is $C_{*}^{\prime }\left(u\right)=1$, the coincidence likelihoods of the random variable $U_{*}$ are $L_{m}\left[U_{*}\right]=1$. A derivation detailed in Appendix A calculates the coincidence likelihoods of the random variable *U*. The derivation implies that the difference $L_{m}\left[U\right]-L_{m}\left[U_{*}\right]$ between the two coincidence likelihoods is the quantity

(10) $$\gamma_{m}\left(Y||X\right)=\int_{R}\left\{{\left[\frac{b\left(r\right)}{a\left(r\right)}\right]}^{m}-1\right\}a\left(r\right)dr.$$

With regard to Equation (10), recall that $a\left(r\right)$ and $b\left(r\right)$ are, respectively, the density functions of the random variables *X* and *Y*.

Evidently, the quantity of Equation (10) can be extended to any real value of the parameter *m*. In what follows, we extend this parameter from the discrete set of values m=2,3,4,⋯ to the continuous range of values m>1.

For the continuous range m>1, a convexity argument that is detailed in Appendix A implies the two following properties. (I) The quantity of Equation (10) is non-negative: $\gamma_{m}\left(Y||X\right)\geq 0$. (II) The quantity of Equation (10) is zero if and only if the random variables *X* and *Y* are equal in law: $\gamma_{m}\left(Y||X\right)=0\Leftrightarrow a\left(r\right)\equiv b\left(r\right)$. Hence, with regard to any parameter value in the continuous range m>1, the following conclusion is attained: the quantity $\gamma_{m}\left(Y||X\right)$ characterizes the case of a zero statistical divergence of the random variable *Y* from the random variable *X*.

The convexity argument that holds for the continuous range m>1 also holds for the continuous range m<0. Shifting from the former range (m>1) to the latter range (m<0) flips the statistical divergence from “*Y* with respect to *X*” to “*X* with respect to *Y*”. Indeed, straightforward calculations that are based on Equation (10) yield the following pair of ‘flipping formulae’. (I) When the parameter is in the continuous range m>1, then $\gamma_{m}\left(X||Y\right)=\gamma_{\left(1-m\right)}\left(Y||X\right)$, in which case (1−m)<0. (II) When the parameter is in the continuous range m<0, then $\gamma_{m}\left(Y||X\right)=\gamma_{\left(1-m\right)}\left(X||Y\right)$, in which case (1−m)>1.

### 2.5. Discussion

The quantity of Equation (10) is intimately related to the Hellinger divergence (see [10,12,26]), as well as to the three divergences that were noted in the introduction: the Kullback–Leibler divergence (also known as “relative entropy”) [13,14,15,16]; the Renyi divergence [17,18,19,20]; and the more general “f-divergence” [17,21,22,23,24,25,26]. These relations are described and discussed hereinafter.

*Hellinger divergence* and *Renyi divergence*. In terms of the quantity of Equation (10), the Hellinger divergence of the random variable *Y* from the random variable *X* admits the formulation $\frac{1}{m-1}\gamma_{m}\left(Y||X\right)$; and the Renyi divergence of the random variable *Y* from the random variable *X* admits the formulation $\frac{1}{m-1}\ln\left[1+\gamma_{m}\left(Y||X\right)\right]$. In both these divergences, the parameter *m* is positive (m>0) and different from one (m≠1).

*Kullback–Leibler divergence*. Setting m=1 in Equation (10), observe that $\gamma_{1}\left(Y||X\right)=0$. In turn, taking the limit m→1—while using L’Hopital’s rule—yields the following limiting result (see Appendix A for the details). The Hellinger divergence $\frac{1}{m-1}\gamma_{m}\left(Y||X\right)$ and the Renyi divergence $\frac{1}{m-1}\ln\left[1+\gamma_{m}\left(Y||X\right)\right]$ converge (as m→1) to the quantity

(11) $$\int_{R}\ln\left[\frac{b\left(r\right)}{a\left(r\right)}\right]b\left(r\right)dr.$$

The quantity appearing in Equation (11) is the Kullback–Leibler divergence of the random variable *Y* from the random variable *X*.

*f-divergence*. The right-hand side of Equation (10) admits the general form

(12) $$\int_{R}\varphi \left[\frac{b\left(r\right)}{a\left(r\right)}\right]a\left(r\right)dr,$$

where $\varphi \left(t\right)$ (t≥0) is a convex function that satisfies the condition $\varphi \left(1\right)=0$. The quantity appearing in Equation (12) is the f-divergence of the random variable *Y* from the random variable *X*. Applying the change in variables $u=A\left(r\right)$ to Equation (12) implies that the f-divergence admits—in terms of the density function $C^{\prime }\left(u\right)$ of Equation (2)—the formulation $\int_{0}^{1}\varphi \left[C^{\prime }\left(u\right)\right]du$.

Different choices of the convex function $\varphi \left(t\right)$ yield different manifestations of the f-divergence (see, for example, [26]). In particular, the choice $\varphi \left(t\right)=t^{m}-1$—with a real parameter *m* that takes values either in the range m>1, or in the range m<0—yields the quantity of Equation (10). The choice $\varphi \left(t\right)=\frac{1}{m-1}\left(t^{m}-1\right)$—with a positive parameter *m* that takes values either in the range m<1, or in the range m>1—yields the Hellinger divergence. And the choice $\varphi \left(t\right)=t\ln\left(t\right)$ yields the Kullback–Leibler divergence.

The convexity argument that was noted in Section 2.4 (with regard to the quantity of Equation (10)) holds for the f-divergence. Namely, the convexity argument implies the two following properties. (I) The f-divergence is non-negative. (II) The f-divergence is zero if and only if the random variables *X* and *Y* are equal in law.

Therefore, via the notion of “coincidence likelihood” (that was described in Section 2.4), the curve $C\left(u\right)$ of Equation (1) leads to Equation (10). In turn, Equation (10) leads to the four aforementioned divergences.

### 2.6. Summary & Implementation

In this section, we have established three different quantities that measure the statistical divergence of the random variable *Y* from the random variable *X*. The random variable *X* takes values in the real range $R=(r_{low},r_{up})$, the random variable *Y* takes values in the real range $\left[r_{low},r_{up}\right]$, and the three quantities are the following.

- $\alpha_{m}\left(Y||X\right)=\left(m+1\right)\mathrm{Pr}\left(X_{1},\cdots ,X_{m}\leq Y\right)-1$, where m=1,2,3,⋯.
- $\beta_{m}\left(Y||X\right)=1-\left(m+1\right)\mathrm{Pr}\left(X_{1},\cdots ,X_{m}>Y\right)$, where m=1,2,3,⋯.
- $\gamma_{m}\left(Y||X\right)=\int_{R}\left\{{\left[\frac{b\left(r\right)}{a\left(r\right)}\right]}^{m}-1\right\}a\left(r\right)dr$, where m>1.

The quantities $\alpha_{m}\left(Y||X\right)$ and $\beta_{m}\left(Y||X\right)$ can be used for any pair of random variables *X* and *Y*. In these quantities, $\left\{X_{1},\cdots ,X_{m}\right\}$ are *m* IID copies of the random variable *X*, and the IID copies are independent of the random variable *Y*. These quantities characterize the case of a zero statistical divergence collectively. Namely, the random variable *Y* is equal in law to the random variable *X* if and only if $\alpha_{m}\left(Y||X\right)=0$ for all *m*, and $\beta_{m}\left(Y||X\right)=0$ for all *m*.

The quantity $\gamma_{m}\left(Y||X\right)$ can be used when the random variable *X* has a density function $a\left(r\right)$ that is positive over the range R, and when the random variable *Y* has a density function $b\left(r\right)$. For any given *m*, this quantity characterizes the case of a zero statistical divergence: the random variable *Y* is equal in law to the random variable *X* if and only if $\gamma_{m}\left(Y||X\right)=0$. The quantity $\gamma_{m}\left(Y||X\right)$ is intimately related to the Hellinger and the Renyi divergences, and it leads to the Kullback–Leibler divergence and to the general f-divergence.

In general, none of the three quantities are symmetric. Namely, in general, the statistical divergence of the random variable *Y* from the random variable *X* is not the same as the statistical divergence of the random variable *X* from the random variable *Y*. Key properties of the three quantities are summarized in Table 1.

The implementation of the three quantities, based on empirical data—be it real-world, experimental, simulated, etc.—is practiced as follows. Firstly, given *n* samples of the random variable *X*, order these samples increasingly: $x_{1}\leq x_{2}\leq \cdots \leq x_{n-1}\leq x_{n}$; in addition, set $x_{0}=r_{low}$ and $x_{n+1}=r_{up}$. Secondly, given a set of samples of the random variable *Y*, calculate the following proportions: $\pi_{i}$ is the proportion of the samples (of the random variable *Y*) that are in the range $\left(x_{i-1},x_{i}\right]$ (i=1,2,⋯,n+1). Thirdly, calculate the three quantities via the following approximation formulae.

- $\alpha_{m}\left(Y||X\right)≃\left(m+1\right)\left[\sum_{i=1}^{n+1}{\left(\frac{i-1/2}{n+1}\right)}^{m}\pi_{i}\right]-1$.
- $\beta_{m}\left(Y||X\right)≃1-\left(m+1\right)\left[\sum_{i=1}^{n+1}{\left(1-\frac{i-1/2}{n+1}\right)}^{m}\pi_{i}\right]$.
- $\gamma_{m}\left(Y||X\right)≃{\left(n+1\right)}^{m-1}\left(\sum_{i=1}^{n+1}\pi_{i}^{m}\right)-1$.

Indeed, based on the empirical data, the estimate of the curve $C\left(u\right)$ is the linear interpolation of the unit-square points $C\left(0\right)=0$ and $C\left(\frac{i}{n+1}\right)=\pi_{1}+\cdots +\pi_{i}$ (i=1,2,⋯,n+1). In turn, the slopes of the piecewise-linear estimate of the curve $C\left(u\right)$ are $C^{\prime }\left(u\right)=\left(n+1\right)\cdot \pi_{i}$ for $\frac{i-1}{n+1}<u<\frac{i}{n+1}$ (i=1,2,⋯,n+1). Consequently, the estimates of the three quantities are given by the above approximation formulae.

## 3. Socioeconomic Application

Consider a positive random variable *W*, whose statistical distribution is governed by the density function $f\left(w\right)$ (0<w<∞). The mean of the random variable *W* is its first moment, $E\left[W\right]=\int_{0}^{\infty }wf\left(w\right)dw$. We assume that the mean is positive and finite, and denote it by μ. This implies that $f_{\$}\left(w\right)=\frac{1}{\mu }wf\left(w\right)$ (0<w<∞) is a density function. We set $W_{\$}$ to be a random variable whose statistical distribution is governed by the density function $f_{\$}\left(w\right)$.

The random variable $W_{\$}$ is positive, and it has a socioeconomic interpretation that is described as follows. Envisage a human society that comprises members with positive personal wealth values, and consider *W* to be the personal wealth of a randomly sampled member of the society. Now, rather than sampling at random a single member of the society, sample at random a single dollar of the society’s overall wealth (i.e., the aggregate of the personal wealth values of all the society’s members). Then, $W_{\$}$ is the personal wealth of the society member to whom the randomly-sampled dollar belongs.

Whereas the random variable *W* has no inherent inclination towards large wealth values, the random variable $W_{\$}$ has such an inclination. Indeed, when a single member of the society is sampled at random, it is exactly as likely for any given rich individual, and for any given poor individual, to be the randomly-sampled member. However, when a single dollar is sampled at random from the society’s overall wealth, it is more likely that this randomly-sampled dollar belongs to a rich member of the society than to a poor member of the society.

This section presents a socioeconomic application of the general framework that was established in Section 2: measuring the statistical divergence of the random variable $W_{\$}$ from the random variable *W*. Throughout this section, $F\left(w\right)=\mathrm{Pr}\left(W\leq w\right)$ (0<w<∞) denotes the distribution function of the random variable *W*. In addition, the density function $f\left(w\right)$ is assumed to be positive, and hence the distribution function $F\left(w\right)$ is increasing, and it has an inverse function $F^{-1}\left(u\right)$ (0<u<1) that is also increasing.

### 3.1. Lorenz Curve

In order to use the framework of Section 2, we set X=W and $Y=W_{\$}$ (the equalities being in law). In turn, the underlying range is the positive half-line $R=\left(0,\infty \right)$ and: the distribution function of *X* is $A\left(r\right)=F\left(r\right)$; the density function of *X* is $a\left(r\right)=f\left(r\right)$; the density function of *Y* is $b\left(r\right)=f_{\$}\left(r\right)=\frac{1}{\mu }rf\left(r\right)$.

Noting that $b\left(r\right)/a\left(r\right)=r/\mu$, Equation (2) implies that the derivative of the curve $C\left(u\right)$ is

(13) $$C^{\prime }\left(u\right)=\frac{1}{\mu }F^{-1}\left(u\right).$$

As the inverse function $F^{-1}\left(u\right)$ is increasing, so is the derivative $C^{\prime }\left(u\right)$, and hence the curve $C\left(u\right)$ is convex. In turn, the convexity of the curve $C\left(u\right)$ implies that its graph resides below the diagonal line of the unit square: $C\left(u\right)<u$ (0<u<1).

The socioeconomic interpretation of the random variable $W_{\$}$ induces a socioeconomic interpretation of the curve $C\left(u\right)$. Indeed, with regard to the underlying human society, the aggregate wealth held by the poor 100u% of the society’s members constitutes $100C\left(u\right)\%$ of the society’s overall wealth. Termed the “Lorenz curve” in honor of the American statistician Max Lorenz [4], the curve $C\left(u\right)$ is widely applied in economics and in the social sciences to investigate wealth and income distributions, as well as to quantify socioeconomic inequality [5,6,7].

As noted after Equation (13), the Lorenz curve is bounded from above by the diagonal line of the unit square: $C\left(u\right)<u$ (0<u<1). In the space of Lorenz curves, the diagonal line characterizes the socioeconomic state of perfect equality: a society in which all the society members have an identical personal wealth value (which is positive).

Evidently, the Lorenz curve is bounded from below by the zero line of the unit square: $C\left(u\right)>0$ (0<u<1). In the space of Lorenz curves, the zero line characterizes the socioeconomic state of perfect inequality: a society in which 0% of the society’s members are in possession of 100% of the society’s overall wealth. The state of perfect inequality is attainable only when the society’s population is infinitely large.

Perhaps the simplest way to envisage the socioeconomic state of perfect inequality is via the following ‘Pharaonic example’. Indeed, consider a Pharaonic society comprising *n* members, in which one single member—the Pharaoh—has personal wealth 1, and all other members have personal wealth 0. Then, the socioeconomic state of perfect inequality is attained in the infinite-population limit n→∞ of the Pharaonic society.

The closer the Lorenz curve is to the diagonal line of the unit square, the more egalitarian the underlying human society. Antithetically, the closer the Lorenz curve is to the zero line of the unit square, the less egalitarian the underlying human society. Hence, the Lorenz curve provides a geometric approach to quantifying socioeconomic inequality.

### 3.2. Gini Index

Named in honor of the Italian economist Corrado Gini [43,44], the “Gini index” is arguably the principal gauge of socioeconomic inequality in economics and in the social sciences [45,46,47,48]. The scientific applications of the Gini index extend well beyond socioeconomic inequality (for recent such applications see [48] and references therein).

In terms of the Lorenz curve, the Gini index is defined as follows: it is twice the area captured between the Lorenz curve and the unit-square’s diagonal line, $2\int_{0}^{1}\left[u-C\left(u\right)\right]du$. The Gini index displays the three following properties. (I) It takes values in the range [0,1]. (II) It attains its lower bound 0 if and only if the underlying human society is in the socioeconomic state of perfect equality. (III) It attains its upper bound 1 if and only if the underlying human society is in the socioeconomic state of perfect inequality.

The definition of the Gini index implies that, with regard to the unit square (in which the graph of the Lorenz curve resides), the Gini index is the difference between the area above the Lorenz curve and the area below the Lorenz curve. Hence, Equation (3) implies that the Gini index is

(14) $$\Delta \left(W_{\$}\left|\right|W\right)=\mathrm{Pr}\left(W_{\$}>W\right)-\mathrm{Pr}\left(W_{\$}\leq W\right).$$

As described above, the random variables *W* and $W_{\$}$ manifest two different wealth-sampling methods. The random variable $W_{\$}$ is inclined towards sampling large wealth values, and the Gini index quantifies this inclination.

### 3.3. Wealth Maxima & Minima

Having addressed the first-order quantity $\Delta \left(W_{\$}\left|\right|W\right)=\alpha_{1}\left(W_{\$}\left|\right|W\right)=\beta_{1}\left(W_{\$}\left|\right|W\right)$ in the previous subsection, we now move from the first order m=1, and address the higher-order quantities $\alpha_{m}\left(Y||X\right)$ and $\beta_{m}\left(Y||X\right)$ (m=2,3,4,⋯). In this subsection, $\left\{W_{1},\cdots ,W_{m+1}\right\}$ denote m+1 IID copies of the random variable W.

A derivation detailed in Appendix A asserts that Equation (4) yields the quantity

(15) $$\alpha_{m}\left(W_{\$}\left|\right|W\right)=\frac{E\left[\max\left\{W_{1},\cdots ,W_{m+1}\right\}\right]-E\left[W\right]}{E\left[W\right]}.$$

Namely, the quantity $\alpha_{m}\left(W_{\$}\left|\right|W\right)$ is the overshoot of the mean of the maximal random variable $\max\left\{W_{1},\cdots ,W_{m+1}\right\}$, measured relative to the mean of the random variable *W*. The quantity $\alpha_{m}\left(W_{\$}\left|\right|W\right)$, via the maximal random variable $\max\left\{W_{1},\cdots ,W_{m+1}\right\}$, sets its focus on the occurrence of large wealth values. It is shown in Appendix A that the quantity $\alpha_{m}\left(W_{\$}\left|\right|W\right)$ takes values in the range [0,m].

A derivation detailed in Appendix A asserts that Equation (5) yields the quantity

(16) $$\beta_{m}\left(W_{\$}\left|\right|W\right)=\frac{E\left[W\right]-E\left[\min\left\{W_{1},\cdots ,W_{m+1}\right\}\right]}{E\left[W\right]}.$$

Namely, the quantity $\beta_{m}\left(W_{\$}\left|\right|W\right)$ is the undershoot of the mean of the minimal random variable $\min\left\{W_{1},\cdots ,W_{m+1}\right\}$, measured relative to the mean of the random variable *W*. The quantity $\beta_{m}\left(W_{\$}\left|\right|W\right)$, via the minimal random variable $\min\left\{W_{1},\cdots ,W_{m+1}\right\}$, sets its focus on the occurrence of small wealth values. It is shown in Appendix A that the quantity $\beta_{m}\left(W_{\$}\left|\right|W\right)$ takes values in the range [0,1].

### 3.4. Gini Index Revisited

As in the previous subsection, in this subsection $\left\{W_{1},\cdots ,W_{m+1}\right\}$ denotes a sample comprising m+1 IID copies of the random variable *W*. The sample’s maximal wealth value is $\max\left\{W_{1},\cdots ,W_{m+1}\right\}$, and the sample’s minimal wealth value is $\min\left\{W_{1},\cdots ,W_{m+1}\right\}$. In turn, the sample’s range is the gap between the sample’s maximal and minimal wealth values, $\max\left\{W_{1},\cdots ,W_{m+1}\right\}-\min\left\{W_{1},\cdots ,W_{m+1}\right\}$.

Summing up Equations (15) and (16), and denoting the sum by $\rho_{m}\left(W_{\$}\left|\right|W\right)=\alpha_{m}\left(W_{\$}\left|\right|W\right)$$+\beta_{m}\left(W_{\$}\left|\right|W\right)$, yields the following quantity:

(17) $$\rho_{m}\left(W_{\$}\left|\right|W\right)=\frac{E\left[\max\left\{W_{1},\cdots ,W_{m+1}\right\}-\min\left\{W_{1},\cdots ,W_{m+1}\right\}\right]}{E\left[W\right]}.$$

Namely, the quantity $\rho_{m}\left(W_{\$}\left|\right|W\right)$ is the mean of the range of the sample $\left\{W_{1},\cdots ,W_{m+1}\right\}$, measured relative to the mean of the random variable *W*. As the quantity $\alpha_{m}\left(W_{\$}\left|\right|W\right)$ takes values in the range [0,m], and as the quantity $\beta_{m}\left(W_{\$}\left|\right|W\right)$ takes values in the range [0,1], the quantity $\rho_{m}\left(W_{\$}\left|\right|W\right)$ takes values in the range [0,m+1].

Consider now the order m=1. With regard to this order, as noted above, the quantities $\alpha_{1}\left(W_{\$}\left|\right|W\right)$ and $\beta_{1}\left(W_{\$}\left|\right|W\right)$ coincide and yield the Gini index $\Delta \left(W_{\$}\left|\right|W\right)$. In turn, the Gini index is the arithmetic average of the quantities $\alpha_{1}\left(W_{\$}\left|\right|W\right)$ and $\beta_{1}\left(W_{\$}\left|\right|W\right)$, and hence $\Delta \left(W_{\$}\left|\right|W\right)=\frac{1}{2}\rho_{1}\left(W_{\$}\left|\right|W\right)$. With regard to the order m=1, the sample is $\left\{W_{1},W_{2}\right\}$ and hence the sample’s range is $\max\left\{W_{1},W_{2}\right\}-\min\left\{W_{1},W_{2}\right\}=\left|W_{1}-W_{2}\right|$. Consequently, Equation (17) implies that

(18) $$\Delta \left(W_{\$}\left|\right|W\right)=\frac{E\left[\left|W_{1}-W_{2}\right|\right]}{2E\left[W\right]}.$$

Namely, the Gini index can be represented as the mean absolute deviation (MAD) of two IID copies of the random variable *W*, measured relative to twice the mean of the random variable *W*.

### 3.5. Wealth Moments

Having addressed the quantities $\alpha_{m}\left(Y||X\right)$ and $\beta_{m}\left(Y||X\right)$, we now turn to address the quantity $\gamma_{m}\left(Y||X\right)$. Setting X=W and $Y=W_{\$}$ (the equalities being in law), a derivation detailed in Appendix A asserts that Equation (10) yields the quantity

(19) $$\gamma_{m}\left(W_{\$}\left|\right|W\right)=\frac{E\left[W^{m}\right]-E{\left[W\right]}^{m}}{E{\left[W\right]}^{m}}.$$

Namely, the quantity $\gamma_{m}\left(W_{\$}\left|\right|W\right)$ is the overshoot of the moment of order *m* of the random variable *W*, measured relative to the $m^{\mathrm{th}}$ power of the mean of the random variable *W*.

For any *m* that is larger than one, a Jensen-inequality argument that is detailed in Appendix A affirms the fact that the quantity $\gamma_{m}\left(W_{\$}\left|\right|W\right)$ is non-negative, $\gamma_{m}\left(W_{\$}\left|\right|W\right)\geq 0$. Moreover, the Jensen-inequality argument asserts that the quantity $\gamma_{m}\left(W_{\$}\left|\right|W\right)$ is zero if and only if the random variable *W* is constant with probability one: $\gamma_{m}\left(W_{\$}\left|\right|W\right)=0\Leftrightarrow \mathrm{Pr}\left(W=\mu \right)=1$. As *m* is larger than one, note that: due to the moment $E\left[W^{m}\right]$, the quantity $\gamma_{m}\left(W_{\$}\left|\right|W\right)$ is sensitive to large wealth values.

For m=2 Equation (19) yields the ratio $\gamma_{2}\left(W_{\$}\left|\right|W\right)=\mathrm{Var}\left[W\right]/E{\left[W\right]}^{2}$, where $\mathrm{Var}\left[W\right]$ is the variance of the random variable *W*. The coefficient of variation (CV) of the random variable *W* is its ‘noise-to-signal’ ratio: the ratio of its standard deviation to its mean, $\sqrt{\mathrm{Var}\left[W\right]}/E\left[W\right]$. Hence, $\gamma_{2}\left(W_{\$}\left|\right|W\right)$ is the squared CV of the random variable *W*.

Setting $X=W_{\$}$ and Y=W (the equalities being in law), a derivation detailed in Appendix A asserts that Equation (10) yields the quantity

(20) $$\gamma_{m}\left(W||W_{\$}\right)=\frac{E\left[W^{1-m}\right]-E{\left[W\right]}^{1-m}}{E{\left[W\right]}^{1-m}}.$$

Namely, the quantity $\gamma_{m}\left(W||W_{\$}\right)$ is the overshoot of the moment of order 1−m of the random variable *W*, measured relative to the ${\left(1-m\right)}^{\mathrm{th}}$ power of the mean of the random variable *W*.

For any *m* that is larger than one, a Jensen-inequality argument that is detailed in Appendix A affirms the fact that the quantity $\gamma_{m}\left(W||W_{\$}\right)$ is non-negative, $\gamma_{m}\left(W||W_{\$}\right)\geq 0$. Moreover, the Jensen inequality argument asserts that the quantity $\gamma_{m}\left(W||W_{\$}\right)$ is zero if and only if the random variable *W* is constant with probability one: $\gamma_{m}\left(W||W_{\$}\right)=0\Leftrightarrow \mathrm{Pr}\left(W=\mu \right)=1$. As *m* is larger than one, note that: due to the moment $E\left[W^{1-m}\right]$, the quantity $\gamma_{m}\left(W||W_{\$}\right)$ is sensitive to small wealth values.

Observing Equations (19) and (20), it is evident that the quantities appearing in these equations are linked by the following relations: $\gamma_{m}\left(W||W_{\$}\right)=\gamma_{1-m}\left(W_{\$}\left|\right|W\right)$, and $\gamma_{m}\left(W_{\$}\left|\right|W\right)=\gamma_{1-m}\left(W||W_{\$}\right)$. These linkages are manifestations of the general ‘flipping formulae’ that were described in Section 2.4.

### 3.6. Inequality Indices

Inequality indices are gauges that quantify the socioeconomic inequality of human societies [1,2,3,8,9]. The aforementioned Gini index is perhaps the best known inequality index. A general inequality index is a ‘socioeconomic score’ that takes values in the range [0,1] and that meets the three following properties. (I) The inequality index yields its lower-bound score 0 if and only if the society is in the socioeconomic state of perfect equality. (II) If the society is in the socioeconomic state of perfect inequality, then the inequality index yields its upper-bound score 1. (III) The inequality index is invariant with respect to the particular currency via which the personal wealth values of the society members are measured (e.g., Dollar or Euro).

With regard to the random variable *W*, the first inequality-index property can be formulated as determinism: the inequality index yields its lower-bound score 0 if and only if the random variable *W* is deterministic, i.e., if and only if it is constant with probability one. In addition, with regard to the random variable *W*, the third inequality-index property can formulated as scale invariance: the inequality index is invariant with respect to the change-of-scale W↦s·W, where *s* is an arbitrary positive scale parameter.

The following quantities all satisfy the first and the third inequality-index properties. the quantities $\alpha_{m}\left(W_{\$}\left|\right|W\right)$ and $\beta_{m}\left(W_{\$}\left|\right|W\right)$ of Equations (15) and (16); the quantity $\rho_{m}\left(W_{\$}\left|\right|W\right)$ of Equation (17); and the quantities $\gamma_{m}\left(W_{\$}\left|\right|W\right)$ and $\gamma_{m}\left(W||W_{\$}\right)$ of Equations (19) and (20). In fact, transformations of these quantities yield inequality indices, and these inequality indices are specified in Table 2. For a comprehensive study of the inequality indices that correspond to the quantities $\alpha_{m}\left(W_{\$}\left|\right|W\right)$, $\beta_{m}\left(W_{\$}\left|\right|W\right)$, and $\rho_{m}\left(W_{\$}\left|\right|W\right)$ the readers are referred to [60]. For a comprehensive study of the inequality indices that correspond to the quantities $\gamma_{m}\left(W_{\$}\left|\right|W\right)$ and $\gamma_{m}\left(W||W_{\$}\right)$, the readers are referred to [60,61].

### 3.7. Summary & Implementation

This section has presented a socioeconomic application of the general framework that was established in Section 2. The application gave rise to well-known socioeconomic notions including the Lorenz curve, the Gini index and its generalizations, and general inequality indices. Underlying this socioeconomic application is the following setting: a human society comprising members with positive personal wealth values.

Wealth is measured via two different statistical sampling methods. On the one hand, a single member of the society is sampled at random, and *W* is the wealth of the randomly-sampled member. On the other hand, a single dollar is sampled at random (from the aggregate wealth of all the society members), and $W_{\$}$ is the wealth of the society member to whom the randomly-sampled dollar belongs. Evidently, the random variable $W_{\$}$ is inclined towards sampling the richer members of the society.

The socioeconomic application focused on measuring the statistical divergence of the random variable $W_{\$}$ from the random variable *W*. This statistical divergence quantifies the extent by which the rich deviate from the rest of the society members. This statistical divergence was shown to be zero if and only if the human society is in the socioeconomic state of perfect equality: all the society members share a common (positive) personal wealth value.

In terms of the random variables *W* and $W_{\$}$, the state of perfect equality is characterized as follows: both the random variables *W* and $W_{\$}$ are equal, with probability one, to a fixed positive wealth value. Thus, from a probabilistic perspective, perfect equality manifests determinism. In turn, the statistical divergence of the random variable $W_{\$}$ from the random variable *W* quantifies the deviation from the ‘deterministic benchmark’.

The statistical divergence of the random variable $W_{\$}$ from the random variable *W* was measured by four quantities: the quantities $\alpha_{m}\left(W_{\$}\left|\right|W\right)$ and $\beta_{m}\left(W_{\$}\left|\right|W\right)$, as well as their sum $\rho_{m}\left(W_{\$}\left|\right|W\right)=\alpha_{m}\left(W_{\$}\left|\right|W\right)+\beta_{m}\left(W_{\$}\left|\right|W\right)$; and the quantity $\gamma_{m}\left(W_{\$}\left|\right|W\right)$. In addition, the statistical divergence of the random variable *W* from the random variable $W_{\$}$ was measured by the quantity $\gamma_{m}\left(W||W_{\$}\right)$. As detailed in Table 2 above, these five quantities can be mapped—via one-to-one transformations—to socioeconomic inequality indices of the underlying human society.

The implementation of the five quantities, based on empirical data—be it real-world, experimental, simulated, etc.—is practiced as follows: Firstly, given *n* samples of the random variable *W*, order these samples increasingly: $w_{1}\leq w_{2}\leq \cdots \leq w_{n-1}\leq w_{n}$. Secondly, calculate the corresponding wealth proportions: $\pi_{i}=w_{i}/\left(w_{1}+\cdots +w_{n}\right)$ (i=1,2,⋯,n). Thirdly, calculate the five quantities via the following approximation formulae.

- $\alpha_{m}\left(W_{\$}\left|\right|W\right)≃\left(m+1\right)\left[\sum_{i=1}^{n}{\left(\frac{i-1/2}{n}\right)}^{m}\pi_{i}\right]-1$.
- $\beta_{m}\left(W_{\$}\left|\right|W\right)≃1-\left(m+1\right)\left[\sum_{i=1}^{n}{\left(1-\frac{i-1/2}{n}\right)}^{m}\pi_{i}\right]$.
- $\rho_{m}\left(W_{\$}\left|\right|W\right)≃\left(m+1\right)\left\{\sum_{i=1}^{n}\left[{\left(\frac{i-1/2}{n}\right)}^{m}+{\left(1-\frac{i-1/2}{n}\right)}^{m}\right]\pi_{i}\right\}$.
- $\gamma_{m}\left(W_{\$}\left|\right|W\right)≃n^{m-1}\left(\sum_{i=1}^{n}\pi_{i}^{m}\right)-1$.
- $\gamma_{m}\left(W||W_{\$}\right)≃n^{-m}\left(\sum_{i=1}^{n}\pi_{i}^{1-m}\right)-1$.

Indeed, based on the empirical data, the estimate of the Lorenz curve $C\left(u\right)$ is the linear interpolation of the unit-square points $C\left(0\right)=0$ and $C\left(\frac{i}{n}\right)=\pi_{1}+\cdots +\pi_{i}$ (i=1,2,⋯,n). In turn, the slopes of the piecewise-linear estimate of the Lorenz curve $C\left(u\right)$ are $C^{\prime }\left(u\right)=n\cdot \pi_{i}$ for $\frac{i-1}{n}<u<\frac{i}{n}$ (i=1,2,⋯,n). Consequently, the estimates of the five quantities are given by the above approximation formulae.

Last but not least, we emphasize that the socioeconomic application presented in this section is not at all confined to the underlying socioeconomic setting. Indeed, the socioeconomic application can be used with regard to any non-negative random variable *W* that has a positive and finite mean [8,9].

## 4. Renewal Application

Consider a positive random variable *T*, whose statistical distribution is governed by the survival function $\bar{F}\left(t\right)=\mathrm{Pr}\left(T>t\right)$ (0<t<∞). The mean of the random variable *T* is given by the integral of its survival function, $E\left[T\right]=\int_{0}^{\infty }\bar{F}\left(t\right)dt$. We assume that the mean is positive and finite, and (as in Section 3) denote it by μ. This implies that $f_{res}\left(t\right)=\frac{1}{\mu }\bar{F}\left(t\right)$ (0<t<∞) is a density function. We set $T_{res}$ to be a random variable whose statistical distribution is governed by the density function $f_{res}\left(t\right)$.

The random variable $T_{res}$ is positive, and it has a renewal interpretation that is described as follows. Consider a renewal process that is generated from the random variable *T* [49,50,51]. Specifically, the renewal process is a sequence of temporal points $0=\tau_{0}<\tau_{1}<\tau_{2}<\tau_{3}<\cdots$ that are termed “renewal epochs”, and that are constructed as follows: the temporal durations between consecutive renewal epochs are IID copies of the random variable *T*. Now, standing at a fixed time point *t*, denote by $\Delta_{t}$ the temporal duration between the time point *t* and the first renewal epoch after the time point *t*. A key result of the theory of renewal processes asserts that [51]: the random variable $\Delta_{t}$ converges in law, in the limit t→∞, to the random variable $T_{res}$—which is termed the “residual lifetime” of the random variable *T*.

The random variables *T* and $T_{res}$ manifest two different observations of the renewal process. To illustrate these different observations, consider the renewal process as manifesting the time epochs at which buses (of a certain bus line) arrive at a given bus station. The random variable *T* manifests the waiting time of a passenger that reaches the bus station just after a bus has left the station. The random variable $T_{res}$ manifests the waiting time of a passenger that reaches the bus station at an arbitrary time point.

This section presents a renewal application of the general framework that was established in Section 2: measuring the statistical divergence of the random variable $T_{res}$ from the random variable *T*. In this section, the survival function $\bar{F}\left(t\right)$ is assumed to be smooth, and $f\left(t\right)=-{\bar{F}}^{\prime }\left(t\right)$ (0<t<∞) denotes the corresponding density function. Namely, $f\left(t\right)$ is the density function of the random variable *T*.

### 4.1. Hazard Rate

The likelihood that the random variable *T* is realized at the positive time point *t* is $f\left(t\right)$. In turn, the conditional likelihood that the random variable *T* is realized at the positive time point *t*—given the information that *T* was not realized up to the time point *t*—is $h\left(t\right)=f\left(t\right)/\bar{F}\left(t\right)$. The conditional likelihood $h\left(t\right)$, as a function of the temporal variable *t*, is termed the “hazard rate” and the “failure rate” of the random variable *T*.

The hazard rate $h\left(t\right)$ characterizes the statistical distribution of the random variable *T*. Indeed, in terms of the hazard rate $h\left(t\right)$, the survival function of the random variable *T* admits the representation $\bar{F}\left(t\right)=\exp\left[-\int_{0}^{t}h\left(u\right)du\right]$. The hazard rate $h\left(t\right)$ is a key statistical tool that is widely applied in survival analysis [62,63,64] and in reliability engineering [65,66,67]. Here, as shall now be shown, the hazard rate $h\left(t\right)$ emerges naturally in the context of the statistical divergence of the random variable $T_{res}$ from the random variable *T*.

In order to use the framework of Section 2, we set X=T and $Y=T_{res}$ (the equalities being in law). In turn, the underlying range is the positive half-line $R=\left(0,\infty \right)$, and: the distribution function of *X* is $A\left(r\right)=F\left(r\right)=1-\bar{F}\left(r\right)$; the density function of *X* is $a\left(r\right)=f\left(r\right)$; the density function of *Y* is $b\left(r\right)=f_{res}\left(r\right)=\frac{1}{\mu }\bar{F}\left(r\right)$.

Noting that $b\left(r\right)/a\left(r\right)=1/\left[\mu \cdot h\left(r\right)\right]$, Equation (2) implies that the derivative of the curve $C\left(u\right)$ is

(21) $$C^{\prime }\left(u\right)=\frac{1}{\mu }\frac{1}{h\left[F^{-1}\left(u\right)\right]}.$$

The curve $C\left(u\right)$ coincides with the diagonal line of the unit square, $C\left(u\right)=u$ (0<u<1), if and only if the curve’s derivative is identically one, $C^{\prime }\left(u\right)=1$ (0<u<1). In turn, Equation (21) implies that the curve’s derivative is identically one if and only if the hazard rate is flat: $h\left(t\right)=1/\mu$ (0<t<∞). This flat hazard rate is equivalent to the exponential survival function $\bar{F}\left(t\right)=\exp\left(-t/\mu \right)$ (0<t<∞).

The above argumentation affirms the well-known fact that the residual lifetime $T_{res}$ (of the random variable *T*) is equal in law to the random variable *T* if and only if this random variable is exponentially distributed. Thus, in effect, the statistical divergence of the random variable $T_{res}$ from the random variable *T* quantifies how “non-exponential” the statistical distribution of the random variable *T* is.

When the random variable *T* is exponentially distributed, then the renewal process that it generates is the Poisson process [52,53,54]. Hence, from a renewal perspective: the statistical divergence of the random variable $T_{res}$ from the random variable *T* quantifies the following: the deviation of the renewal process that is generated by the random variable *T* from the ‘Poisson benchmark’.

### 4.2. Increasing and Decreasing Hazard Rates

In reliability engineering, two particular classes of random variables are distinguished as significantly important [65]. One is the increasing failure rate (IFR) class, which comprises all positive random variables whose hazard rates are increasing functions. The other is the decreasing failure rate (DFR) class, which comprises all positive random variables whose hazard rates are decreasing functions.

The IFR class is used to model the lifetimes of systems that age with time. Namely, a system is aging if the likelihood that the system will fail grows as the age of the system grows. Aging systems are all around us, and examples of such systems include buildings, infrastructures, machines, cars, ships, airplanes, and even our very own human bodies.

If the random variable *T* belongs to the IFR class, then its hazard rate $h\left(t\right)$ is an increasing function. In this IFR scenario, Equation (21) implies that the derivative $C^{\prime }\left(u\right)$ is decreasing, and hence the curve $C\left(u\right)$ is concave. In turn, the concavity of the curve $C\left(u\right)$ implies that its graph resides above the diagonal line of the unit square: $C\left(u\right)>u$ (0<u<1).

The DFR class is used to model the lifetimes of phenomena that anti-age with time. Namely, a phenomenon is anti-aging if the likelihood that the phenomenon will cease diminishes as the age of the phenomenon grows [68]. Anti-aging phenomena are encountered in our culture and in our technologies, e.g., the symphonies of Beethoven, the writings of Shakespeare, the Georgian calendar, the English alphabet, cutlery, and the wheel. Indeed, the longer we listen to Beethoven and the longer we use cutlery, the greater the likelihood that we shall keep on doing so.

If the random variable *T* belongs to the DFR class, then its hazard rate $h\left(t\right)$ is a decreasing function. In this DFR scenario, Equation (21) implies that the derivative $C^{\prime }\left(u\right)$ is increasing, and hence the curve $C\left(u\right)$ is convex. In turn, the convexity of the curve $C\left(u\right)$ implies that its graph resides below the diagonal line of the unit square: $C\left(u\right)<u$ (0<u<1).

As noted in Section 2.1, the curve $C\left(u\right)$ splits the unit square into two sets: the square’s points that are above the curve, and the square’s points that are below the curve. Moreover, Equation (3) asserts that the difference between the area of the upper set and the area of the lower set is the quantity

(22) $$\Delta \left(T_{res}\left|\right|T\right)=\mathrm{Pr}\left(T_{res}>T\right)-\mathrm{Pr}\left(T_{res}\leq T\right).$$

The quantity $\Delta \left(T_{res}\left|\right|T\right)$ takes values in the range [−1,1], and for the IFR and DFR classes, it gauges the “deviation from exponentiality”.

Indeed, if the random variable *T* belongs to the IFR class, then the quantity $\Delta \left(T_{res}\left|\right|T\right)$ takes values in the range [−1,0], and it displays the following properties. (I) The quantity $\Delta \left(T_{res}\left|\right|T\right)$ attains its upper bound 0 if and only if the random variable *T* is exponentially distributed. (II) The smaller the quantity $\Delta \left(T_{res}\left|\right|T\right)$, the more “non-exponential” the statistical distribution of the random variable *T* is.

Similarly, if the random variable *T* belongs to the DFR class, then the quantity $\Delta \left(T_{res}\left|\right|T\right)$ takes values in the range [0,1], and it displays the following properties. (I) The quantity $\Delta \left(T_{res}\left|\right|T\right)$ attains its lower bound 0 if and only if the random variable *T* is exponentially distributed. (II) The larger the quantity $\Delta \left(T_{res}\left|\right|T\right)$, the more “non-exponential” the statistical distribution of the random variable *T* is.

In statistical physics, the exponential distribution is the paradigmatic model for ‘regular’ relaxation. The IFR and DFR classes offer general models for ‘anomalous’ relaxation [55,56,57,58]. Specifically, the IFR class is a general model for super-exponential relaxation, and the IFR class is a general model for sub-exponential relaxation. In turn, the quantity $\Delta \left(T_{res}\left|\right|T\right)$ quantifies the deviation of anomalous relaxation—super-exponential and sub-exponential—from the ‘regular-relaxation benchmark’.

### 4.3. Weibull Example

To illustrate the quantity $\Delta \left(T_{res}\left|\right|T\right)$ in the context of the IFR and DFR classes, consider the example of a Weibull-distributed random variable *T* [69,70,71]. This example is characterized by the survival function $\bar{F}\left(t\right)=\exp\left[-{\left(t/s\right)}^{\epsilon }\right]$ (0<t<∞), where *s* is a positive scale parameter, and where ϵ is a positive exponent. Equivalently, this example is characterized by the hazard rate $h\left(t\right)=ct^{\epsilon -1}$ (0<t<∞), where $c=\epsilon /s^{\epsilon }$ is a positive coefficient.

The Weibull hazard rate exhibits the following behaviors. For exponent values ϵ<1 the hazard rate is decreasing, and hence the random variable *T* belongs to the DFR class. At the exponent value ϵ=1 the hazard rate is flat, and hence the random variable *T* is exponentially distributed—with a mean that equals the scale parameter, μ=s. For exponent values ϵ>1, the hazard rate is increasing, and hence the random variable *T* belongs to the IFR class.

A calculation using a general result to be presented below (Equation (25)) yields—for the Weibull-distributed random variable *T*—the quantity

(23) $$\Delta \left(T_{res}\left|\right|T\right)=1-2^{1-\frac{1}{\epsilon }}.$$

Note that the right-hand side of Equation (23) depends only on the Weibull exponent ϵ (i.e., it does not depend on the scale parameter *s*). Denoting the right-hand side of Equation (23) by $g\left(\epsilon \right)$, this function of the Weibull exponent ϵ decreases from the level $\lim_{\epsilon \to 0}g\left(\epsilon \right)=1$ to the level $\lim_{\epsilon \to \infty }g\left(\epsilon \right)=-1$. In addition, this function passes through the origin at the exponent value one, $g\left(1\right)=0$.

Thus, for the Weibull-distributed random variable *T*, the quantity $\Delta \left(T_{res}\left|\right|T\right)$ exhibits the following behaviors. In the DFR range (ϵ<1), the quantity $\Delta \left(T_{res}\left|\right|T\right)$ is positive, and this quantity attains its upper bound 1 in the Weibull limit ϵ→0. The quantity $\Delta \left(T_{res}\left|\right|T\right)$ is zero if and only if the Weibull exponent ϵ is one, which characterizes the case of an exponentially distributed random variable *T*. In the IFR range (ϵ>1), the quantity $\Delta \left(T_{res}\left|\right|T\right)$ is negative, and this quantity attains its lower bound −1 in the Weibull limit ϵ→∞.

As noted in Section 4.2, the exponential distribution is the paradigmatic model for ‘regular’ relaxation in statistical physics. The paradigmatic model for ‘anomalous’ relaxation is the Weibull distribution [55,56,57,58]. The Weibull distribution spans sub-exponential anomalous relaxation (the DFR range ϵ<1), super-exponential anomalous relaxation (the IFR range ϵ>1), and ‘regular’ exponential relaxation (the ϵ=1 boundary, which separates the DFR and the IFR ranges). So, with regard to the Weibull model of anomalous relaxation, the quantity $\Delta \left(T_{res}\left|\right|T\right)$ of Equation (23) quantifies the deviation from the ‘regular-relaxation benchmark’.

### 4.4. Duration Maxima and Minima

Having addressed and exemplified the first-order quantity $\Delta \left(T_{res}\left|\right|T\right)=\alpha_{1}\left(T_{res}\left|\right|T\right)=\beta_{1}\left(T_{res}\left|\right|T\right)$ in the previous subsections, we now elevate from the first order m=1, and address the higher-order quantities $\alpha_{m}\left(T_{res}\left|\right|T\right)$ and $\beta_{m}\left(T_{res}\left|\right|T\right)$ (m=2,3,4,⋯). In this subsection, $\left\{T_{1},\cdots ,T_{m+1}\right\}$ denote m+1 IID copies of the random variable T.

A derivation detailed in Appendix A asserts that Equation (4) yields the quantity

(24) $$\alpha_{m}\left(T_{res}\left|\right|T\right)=\left(m+1\right)\frac{E\left[\max\left\{T_{1},\cdots ,T_{m+1}\right\}\right]-E\left[\max\left\{T_{1},\cdots ,T_{m}\right\}\right]}{E\left[T\right]}-1.$$

The quantity $\alpha_{m}\left(T_{res}\left|\right|T\right)$ is based on the difference between the mean of the maximal random variable $\max\left\{T_{1},\cdots ,T_{m+1}\right\}$ and the mean of the maximal random variable $\max\left\{T_{1},\cdots ,T_{m}\right\}$. The quantity $\alpha_{m}\left(T_{res}\left|\right|T\right)$, via the maximal random variables $\max\left\{T_{1},\cdots ,T_{m+1}\right\}$ and $\max\left\{T_{1},\cdots ,T_{m}\right\}$, sets its focus on the occurrence of large values of the duration *T*. As noted in Section 2.2, the quantity $\alpha_{m}\left(T_{res}\left|\right|T\right)$ takes values in the range [−1,m].

A derivation detailed in Appendix A asserts that Equation (5) yields the quantity

(25) $$\beta_{m}\left(T_{res}\left|\right|T\right)=1-\left(m+1\right)\frac{E\left[\min\left\{T_{1},\cdots ,T_{m+1}\right\}\right]}{E\left[T\right]}.$$

The quantity $\beta_{m}\left(T_{res}\left|\right|T\right)$ is based on the mean of the minimal random variable $\min\left\{T_{1},\cdots ,T_{m+1}\right\}$. The quantity $\beta_{m}\left(T_{res}\left|\right|T\right)$, via the minimal random variable $\min\left\{T_{1},\cdots ,T_{m+1}\right\}$, sets its focus on the occurrence of small values of the duration *T*. As noted in Section 2.2, the quantity $\beta_{m}\left(T_{res}\left|\right|T\right)$ takes values in the range [−m,1].

### 4.5. Duration-Wealth Linkages

This section addresses the statistical divergence of the random variable $T_{res}$ from the random variable *T*, where $T_{res}$ is a random variable whose statistical distribution is governed by the density function $f_{res}\left(t\right)=\frac{1}{\mu }\bar{F}\left(t\right)$ (0<t<∞). The previous section addressed the statistical divergence of the random variable $T_{\$}$ from the random variable *T*, where $T_{\$}$ is a random variable whose statistical distribution is governed by the density function $f_{\$}\left(t\right)=\frac{1}{\mu }tf\left(t\right)$ (0<t<∞).

In this subsection we will show that the quantities $\alpha_{m}\left(T_{res}\left|\right|T\right)$ and $\beta_{m}\left(T_{res}\left|\right|T\right)$ of this section are intimately linked to the quantities $\alpha_{m}\left(T_{\$}\left|\right|T\right)$ and $\beta_{m}\left(T_{\$}\left|\right|T\right)$ of the previous section. Indeed, observing Equation (24) on the one hand, and Equation (15) on the other hand, it follows that

(26) $$\alpha_{m}\left(T_{res}\left|\right|T\right)=\left(m+1\right)\left[\alpha_{m}\left(T_{\$}\left|\right|T\right)-\alpha_{m-1}\left(T_{\$}\left|\right|T\right)\right]-1.$$

Moreover, observing Equation (25) on the one hand, and Equation (16) on the other hand, it follows that

(27) $$\beta_{m}\left(T_{res}\left|\right|T\right)=\left(m+1\right)\beta_{m}\left(T_{\$}\left|\right|T\right)-m.$$

In particular, setting m=1 in Equations (26) and (27) yields

(28) $$\Delta \left(T_{res}\left|\right|T\right)=2\Delta \left(T_{\$}\left|\right|T\right)-1.$$

Specifically, Equation (28) follows from Equations (26) and (27) by noting that $\alpha_{0}\left(T_{\$}\left|\right|T\right)=0$ (indeed, setting m=0 in Equation (15) yields $\alpha_{0}\left(T_{\$}\left|\right|T\right)=0$), and, by using Equation (6) (with regard to the divergence of $T_{res}$ from *T*, and with regard to the divergence of $T_{\$}$ from *T*). Recall that the quantity $\Delta \left(T_{\$}\left|\right|T\right)$ that appears on the right-hand side of Equation (28) is the Gini index of the random variable *T*.

As described in the opening of this section, the random variable $T_{res}$ has a renewal interpretation. And, as described in the opening of the previous section, the random variable $T_{\$}$ has a wealth interpretation. In fact, the random variable $T_{\$}$ also has a renewal interpretation which is described as follows.

Consider a renewal process that is generated from the random variable *T* (as detailed in the opening of this section). Standing at a fixed positive time point *t*, denote by $C_{t}$ the temporal duration of the renewal interval that ‘covers’ the time point *t*. Namely, $C_{t}$ is the temporal duration between the following renewal epochs: the last renewal epoch before the time point *t*, and the first renewal epoch after the time point *t*. A key result of the theory of renewal processes asserts that [51]: the random variable $C_{t}$ converges in law, in the limit t→∞, to the random variable $T_{\$}$. In this renewal context, the random variable $T_{\$}$ is termed the “total lifetime” of the random variable *T*.

The random variables *T* and $T_{\$}$ manifest two different observations of the renewal process. To illustrate these different observations, consider (as in the opening of this section) the renewal process as manifesting the time epochs at which buses (of a certain bus line) arrive at a given bus station. The random variable *T* manifests the waiting time between consecutive bus arrivals—as observed by a passenger that reaches the bus station just after a bus has left the station. The random variable $T_{\$}$ manifests the waiting time between consecutive bus arrivals—as observed by a passenger that reaches the bus station at an arbitrary time point.

### 4.6. Inequality Indices Revisited

Equations (26) and (27) established linkages between the quantities $\alpha_{m}\left(T_{res}\left|\right|T\right)$ and $\beta_{m}\left(T_{res}\left|\right|T\right)$ of this section, and the quantities $\alpha_{m}\left(T_{\$}\left|\right|T\right)$ and $\beta_{m}\left(T_{\$}\left|\right|T\right)$ of the previous section. In the previous section, it was shown that transformations of the quantities $\alpha_{m}\left(T_{\$}\left|\right|T\right)$ and $\beta_{m}\left(T_{\$}\left|\right|T\right)$ are inequality indices of the random variable *T*. Thus, the following question arises naturally: are there transformations of the quantities $\alpha_{m}\left(T_{res}\left|\right|T\right)$ and $\beta_{m}\left(T_{res}\left|\right|T\right)$ that are also inequality indices? This subsection shall answer the question affirmatively.

The following transformation of the quantity $\alpha_{m}\left(T_{res}\left|\right|T\right)$ is an inequality index:

(29) $$\frac{\alpha_{m}\left(T_{res}\left|\right|T\right)+1}{m+1}=\frac{E\left[\max\left\{T_{1},\cdots ,T_{m+1}\right\}\right]-E\left[\max\left\{T_{1},\cdots ,T_{m}\right\}\right]}{E\left[T\right]}.$$

Indeed, setting X=T and $Y=T_{res}$ in Equation (4), it follows that the term appearing in Equation (29) is the probability $\mathrm{Pr}\left(X_{1},\cdots ,X_{m}\leq Y\right)$—which, of course, takes values in the range $\left[0,1\right]$. The right-hand side of Equation (29) attains the lower bound 0 if and only if the random variable *T* is constant with probability one. In addition, the right-hand side of Equation (29) is invariant with respect to changes-of-scale of the random variable *T*.

The following transformation of the quantity $\beta_{m}\left(T_{res}\left|\right|T\right)$ is an inequality index:

(30) $$\frac{\beta_{m}\left(T_{res}\left|\right|T\right)+m}{m+1}=\frac{E\left[T\right]-E\left[\min\left\{T_{1},\cdots ,T_{m+1}\right\}\right]}{E\left[T\right]}.$$

Indeed, setting X=T and $Y=T_{res}$ in Equation (5), it follows that the term appearing in Equation (30) is the probability $\mathrm{Pr}\left(X_{1},\cdots ,X_{m}>Y\right)$, which, of course, takes values in the range $\left[0,1\right]$. The right-hand side of Equation (30) attains the lower bound 0 if and only if the random variable *T* is constant with probability one. Moreover, the right-hand side of Equation (30) is invariant with respect to changes-of-scale of the random variable *T*.

Equation (27) implies that the left-hand side of Equation (30) is equal to the quantity $\beta_{m}\left(T_{\$}\left|\right|T\right)$. In the previous section, we saw that the quantity $\beta_{m}\left(T_{\$}\left|\right|T\right)$ is an inequality index, and hence Equation (30) does not yield a ‘new’ inequality index. On the other hand, Equation (29) does yield a ‘new’ inequality index, i.e., an inequality index that was not encountered in the previous section.

### 4.7. Summary and Implementation

This section has presented a renewal application of the general framework that was established in Section 2. Underlying the renewal application is the following setting: a renewal process whose inter-renewal durations—i.e., the temporal durations between the process’ consecutive renewal epochs—are IID copies of the random variable *T*.

The waiting-time till the next renewal epoch was observed via two different temporal perspectives. On the one hand, an observer was placed right after a renewal epoch; the waiting time of this observer was the random variable *T*. On the other hand, an observer was placed at an arbitrary time point; the waiting time of this observer was the random variable $T_{res}$—the “residual lifetime” of the random variable *T*.

The renewal application focused on measuring the statistical divergence of the random variable $T_{res}$ from the random variable *T*. In effect, this statistical divergence quantifies the extent by which the distribution of the random variable *T* deviates from the exponential distribution. Indeed, the statistical divergence of $T_{res}$ from *T* was shown to be zero if and only if the random variables *T* and $T_{res}$ share a common exponential distribution.

The renewal process whose inter-renewal durations are exponentially distributed is the Poisson process. Hence, from a renewal perspective: the statistical divergence of $T_{res}$ from *T* quantifies the deviation of renewal processes from the ‘Poisson benchmark’. In statistical physics the exponential distribution is the paradigmatic model of ‘regular’ relaxation. Hence, from a statistical-physics perspective: the statistical divergence of $T_{res}$ from *T* quantifies the deviation of anomalous relaxation from the ‘regular-relaxation benchmark’.

The statistical divergence of the random variable $T_{res}$ from the random variable *T* was measured using two quantities, $\alpha_{m}\left(T_{res}\left|\right|T\right)$ and $\beta_{m}\left(T_{res}\left|\right|T\right)$. As shown above, these two quantities can be mapped—via one-to-one transformations—to inequality indices of the random variable *T*. The measurement of the statistical divergence of the random variable $T_{res}$ from the random variable *T* by the quantity $\gamma_{m}\left(T_{res}\left|\right|T\right)$ is detailed in Appendix A. Also detailed in Appendix A is the measurement of the statistical divergence of the random variable *T* from the random variable $T_{res}$ via the quantity $\gamma_{m}\left(T||T_{res}\right)$.

The implementation of the aforementioned quantities, based on empirical data—be it real-world, experimental, simulated, etc.—is practiced as follows. Firstly, given *n* samples of the random variable *T*, order these samples increasingly: $t_{1}\leq t_{2}\leq \cdots \leq t_{n-1}\leq t_{n}$. Secondly, set $\pi_{0}=0$ and calculate the following proportions: $\pi_{i}=t_{i}/\left(t_{1}+\cdots +t_{n}\right)$ (i=1,2,⋯,n). Thirdly, calculate the quantities via the following approximation formulae.

- $\alpha_{m}\left(T_{res}\left|\right|T\right)≃\left(m+1\right)\left[\sum_{i=1}^{n}{\left(\frac{i-1/2}{n}\right)}^{m}\left(n-i+1\right)\left(\pi_{i}-\pi_{i-1}\right)\right]-1$.
- $\beta_{m}\left(T_{res}\left|\right|T\right)≃1-\left(m+1\right)\left[\sum_{i=1}^{n}{\left(1-\frac{i-1/2}{n}\right)}^{m}\left(n-i+1\right)\left(\pi_{i}-\pi_{i-1}\right)\right]$.
- $\gamma_{m}\left(T_{res}\left|\right|T\right)≃n^{m-1}\left\{\sum_{i=1}^{n}{\left[\left(n-i+1\right)\left(\pi_{i}-\pi_{i-1}\right)\right]}^{m}\right\}-1$.

Indeed, based on the empirical data, it is shown in Appendix A that the estimate of the curve $C\left(u\right)$ is the linear interpolation of the unit-square points $C\left(0\right)=0$ and $C\left(\frac{i}{n}\right)=\left(\pi_{1}+\cdots +\pi_{i}\right)+\left(n-i\right)\pi_{i}$ (i=1,2,⋯,n). In turn, the slopes of the piecewise-linear estimate of the curve $C\left(u\right)$ are $C^{\prime }\left(u\right)=n\left(n-i+1\right)\left(\pi_{i}-\pi_{i-1}\right)$ for $\frac{i-1}{n}<u<\frac{i}{n}$ (i=1,2,⋯,n). Consequently, the estimates of the aforementioned quantities are given by the above approximation formulae. An alternative way to implement the quantities $\alpha_{m}\left(T_{res}\left|\right|T\right)$ and $\beta_{m}\left(T_{res}\left|\right|T\right)$ is the following: use Equations (26) and (27) to represent these quantities in terms of the corresponding quantities of Section 3, and use the approximation formulae of Section 3.7 with regard to the latter quantities.

## 5. Conclusions

This paper addressed the topic of statistical divergence. To that end, a pair of random variables, which take values in a common real range, were considered: a ‘benchmark’ random variable *X*, and a random variable *Y* of interest. The focus was put on gauging the distance of the statistical distribution of *Y* from the statistical distribution of *X*.

A general framework of statistical divergence was established in Section 2, and was summarized in Section 2.6. The general framework was constructed in a simple and transparent fashion, and the gauges it yielded included the Hellinger divergence, the Renyi divergence, the Kullback–Leibler divergence, and the f-divergence. Two applications of the general framework were then presented.

The first application was to the topic of socioeconomic inequality. This application was detailed in Section 3, and was summarized in Section 3.7. The fruits that this application yielded included the Lorenz curve, socioeconomic inequality indices, the Gini index, and generalizations of the Gini index.

The second application was to the topic of renewal processes. This application was detailed in Section 4, and was summarized in Section 4.7. The fruits that this application yielded included gauges that quantify the divergence of renewal processes from the Poisson process, gauges that quantify the divergence of anomalous relaxation from regular relaxation, and further generalizations of the Gini index.

Empirical applications of the general framework are beyond the scope of this paper. Each of the aforementioned summary subsections provided ‘implementation formulae’. Namely, with regard to given empirical data—be it real-world, experimental, simulated, etc.—the implementation formulae specify how to calculate the gauges that were established and presented here.

Theoretically, this paper offers its readers a transparent and rather general path to statistical divergence, paths thereof to further topics, and deep linkages between the different (and seemingly unrelated) topics addressed. Practically, this paper offers its readers potent gauges of statistical divergence, and explicit formulae that specify how to implement the gauges. Theoretically and practically alike, this paper provides a wide and multi-disciplinary perspective on statistical divergence.

## Figures and Tables

**Table 1:** Key properties of the measures established in Section 2—the quantities $\alpha_{m}\left(Y||X\right)$, $\beta_{m}\left(Y||X\right)$, and $\gamma_{m}\left(Y||X\right)$. For each quantity, the table specifies the values of the underlying parameter *m*, the range of the quantity, and the characterization of the case where the random variable *Y* is equal in law to the random variable *X*. For the quantities $\alpha_{m}\left(Y||X\right)$ and $\beta_{m}\left(Y||X\right)$, the table also specifies the characterization of the following extreme cases: the random variable *Y* is equal, with probability one (w.p. 1), to the lower-bound value $r_{low}$; the random variable *Y* is equal, with probability one (w.p. 1), to the upper-bound value $r_{up}$.

Quantity	$\alpha_{m}\left(Y||X\right)$	$\beta_{m}\left(Y||X\right)$	$\gamma_{m}\left(Y||X\right)$
Parameter	m=1,2,3,⋯	m=1,2,3,⋯	m>1
Range	$-1\leq \alpha_{m}\left(Y||X\right)\leq m$	$-m\leq \beta_{m}\left(Y||X\right)\leq 1$	$0\leq \gamma_{m}\left(Y||X\right)<\infty$
$\begin{array}{c} Y=X\\ (\mathrm{in}\;\mathrm{law}) \end{array}$	$\begin{array}{c} \alpha_{m}\left(Y||X\right)=0\\ \;(\mathrm{all}\;m) \end{array}$	$\begin{array}{c} \beta_{m}\left(Y||X\right)=0\\ \;(\mathrm{all}\;m) \end{array}$	$\begin{array}{c} \gamma_{m}\left(Y||X\right)=0\\ \;(\mathrm{any}\;m) \end{array}$
$\begin{array}{c} Y=r_{low}\\ (w.p.\;1) \end{array}$	$\alpha_{m}\left(Y||X\right)=-1$	$\beta_{m}\left(Y||X\right)=-m$	——
$\begin{array}{c} Y=r_{up}\\ (w.p.\;1) \end{array}$	$\alpha_{m}\left(Y||X\right)=m$	$\beta_{m}\left(Y||X\right)=1$	——

**Table 2:** Inequality indices that are obtained via transformations of the quantities presented and discussed in Section 3. For each quantity, the table specifies the transformation and the resulting inequality index. The values of the underlying parameter *m* are as in Table 1: m=1,2,3,⋯ for the quantities $\alpha_{m}\left(W_{\$}\left|\right|W\right)$ and $\beta_{m}\left(W_{\$}\left|\right|W\right)$, as well as for the quantity $\rho_{m}\left(W_{\$}\left|\right|W\right)$; and m>1 for the quantities $\gamma_{m}\left(W_{\$}\left|\right|W\right)$ and $\gamma_{m}\left(W||W_{\$}\right)$.

Quantity	Transformation	Inequality Index
$\alpha_{m}\left(W_{\$}\left|\right|W\right)$	$\frac{1}{m}\alpha_{m}\left(W_{\$}\left|\right|W\right)$	$\frac{E\left[\max\left\{W_{1},\cdots ,W_{m+1}\right\}\right]-E\left[W\right]}{mE\left[W\right]}$
$\beta_{m}\left(W_{\$}\left|\right|W\right)$	$\beta_{m}\left(W_{\$}\left|\right|W\right)$	$\frac{E\left[W\right]-E\left[\min\left\{W_{1},\cdots ,W_{m+1}\right\}\right]}{E\left[W\right]}$
$\rho_{m}\left(W_{\$}\left|\right|W\right)$	$\frac{1}{m+1}\rho_{m}\left(W_{\$}\left|\right|W\right)$	$\frac{E\left[\max\left\{W_{1},\cdots ,W_{m+1}\right\}-\min\left\{W_{1},\cdots ,W_{m+1}\right\}\right]}{\left(m+1\right)E\left[W\right]}$
ine $\gamma_{m}\left(W_{\$}\left|\right|W\right)$	$\frac{\gamma_{m}\left(W_{\$}\left|\right|W\right)}{1+\gamma_{m}\left(W_{\$}\left|\right|W\right)}$	$1-\frac{E{\left[W\right]}^{m}}{E\left[W^{m}\right]}$
$\gamma_{m}\left(W||W_{\$}\right)$	$\frac{\gamma_{m}\left(W||W_{\$}\right)}{1+\gamma_{m}\left(W||W_{\$}\right)}$	$1-\frac{E{\left[W\right]}^{1-m}}{E\left[W^{1-m}\right]}$

## Data Availability

No new data were created or analyzed in this study.

## Appendix A

This section provides detailed derivations of various results that were stated along the paper.

### Appendix A.1. Derivations: Section 2

#### Appendix A.1.1. The Area under the Curve *C*(*u*)

Using the change of variables $u=A\left(r\right)$, Equation (1) implies that

(A1) $$\int_{0}^{1}C\left(u\right)du=\int_{0}^{1}B\left[A^{-1}\left(u\right)\right]du=\int_{R}B\left(r\right)a\left(r\right)dr.$$

For *X* and *Y* that are mutually independent, conditioning on the value of *X* implies that

(A2) $$\left.\begin{array}{c} \mathrm{Pr}\left(Y\leq X\right)=\int_{R}\mathrm{Pr}\left(Y\leq r|X=r\right)a\left(r\right)dr\\ \;\\ =\int_{R}\mathrm{Pr}\left(Y\leq r\right)a\left(r\right)dr=\int_{R}B\left(r\right)a\left(r\right)dr. \end{array}\right.$$

Combined together, Equations (A1) and (A2) imply that

(A3) $$\int_{0}^{1}C\left(u\right)du=\mathrm{Pr}\left(Y\leq X\right).$$

#### Appendix A.1.2. The Moment **E**[*U^m^*]

The density function of Equation (2), and the change of variables $u=A\left(r\right)$, imply that

(A4) $$\left.\begin{array}{c} E\left[U^{m}\right]=\int_{0}^{1}u^{m}C^{\prime }\left(u\right)du\\ \;\\ =\int_{0}^{1}u^{m}\frac{b\left[A^{-1}\left(u\right)\right]}{a\left[A^{-1}\left(u\right)\right]}du=\int_{R}A{\left(r\right)}^{m}\frac{b\left(r\right)}{a\left(r\right)}a\left(r\right)dr\\ \;\\ =\int_{R}A{\left(r\right)}^{m}b\left(r\right)dr. \end{array}\right.$$

For $\left\{X_{1},\cdots ,X_{m}\right\}$ that are IID copies of the random variable *X*, which are independent of the random variable *Y*, conditioning on the value of *Y* implies that

(A5) $$\left.\begin{array}{c} \mathrm{Pr}\left(X_{1},\cdots ,X_{m}\leq Y\right)=\int_{R}\mathrm{Pr}\left(X_{1},\cdots ,X_{m}\leq r|Y=r\right)b\left(r\right)dr\\ \;\\ =\int_{R}\mathrm{Pr}\left(X_{1},\cdots ,X_{m}\leq r\right)b\left(r\right)dr\\ \;\\ =\int_{R}\left[\mathrm{Pr}\left(X_{1}\leq r\right)\cdots \mathrm{Pr}\left(X_{m}\leq r\right)\right]b\left(r\right)dr\\ \;\\ =\int_{R}A{\left(r\right)}^{m}b\left(r\right)dr. \end{array}\right.$$

Combined together, Equations (A4) and (A5) imply that

(A6) $$E\left[U^{m}\right]=\mathrm{Pr}\left(X_{1},\cdots ,X_{m}\leq Y\right).$$

#### Appendix A.1.3. The Moment **E**[(1 − *U*)*_m_*]

Denote by $\bar{A}\left(r\right)=\mathrm{Pr}\left(X>r\right)$ (r∈R) the survival function of the random variable *X*, and note that $\bar{A}\left(r\right)=1-A\left(r\right)$. The density function of Equation (2), and the change of variables $u=A\left(r\right)$, imply that

(A7) $$\left.\begin{array}{c} E\left[{\left(1-U\right)}^{m}\right]=\int_{0}^{1}{\left(1-u\right)}^{m}C^{\prime }\left(u\right)du\\ \;\\ =\int_{0}^{1}{\left(1-u\right)}^{m}\frac{b\left[A^{-1}\left(u\right)\right]}{a\left[A^{-1}\left(u\right)\right]}du=\int_{R}{\left[1-A\left(r\right)\right]}^{m}\frac{b\left(r\right)}{a\left(r\right)}a\left(r\right)dr\\ \;\\ =\int_{R}\bar{A}{\left(r\right)}^{m}b\left(r\right)dr. \end{array}\right.$$

For $\left\{X_{1},\cdots ,X_{m}\right\}$ that are IID copies of the random variable *X*, and that are independent of the random variable *Y*, conditioning on the value of *Y* implies that

(A8) $$\left.\begin{array}{c} \mathrm{Pr}\left(X_{1},\cdots ,X_{m}>Y\right)=\int_{R}\mathrm{Pr}\left(X_{1},\cdots ,X_{m}>r|Y=r\right)b\left(r\right)dr\\ \;\\ =\int_{R}\mathrm{Pr}\left(X_{1},\cdots ,X_{m}>r\right)b\left(r\right)dr\\ \;\\ =\int_{R}\left[\mathrm{Pr}\left(X_{1}>r\right)\cdots \mathrm{Pr}\left(X_{m}>r\right)\right]b\left(r\right)dr\\ \;\\ \int_{R}\bar{A}{\left(r\right)}^{m}b\left(r\right)dr. \end{array}\right.$$

Combined together, Equations (A7) and (A8) imply that

(A9) $$E\left[{\left(1-U\right)}^{m}\right]=\mathrm{Pr}\left(X_{1},\cdots ,X_{m}>Y\right).$$

#### Appendix A.1.4. Derivation of Equations (7) and (8)

Consider a smooth and bounded function $\psi \left(u\right)$ that is defined over the unit interval (0<u<1). As $C^{\prime }\left(u\right)$ is the density function of the random variable *U*, and as $C_{*}^{\prime }\left(u\right)=1$ is the density function of the random variable $U_{*}$, we have

(A10) $$\left.\begin{array}{c} E\left[\psi \left(U\right)\right]-E\left[\psi \left(U_{*}\right)\right]\\ \;\\ =\int_{0}^{1}\psi \left(u\right)C^{\prime }\left(u\right)du-\int_{0}^{1}\psi \left(u\right)C_{*}^{\prime }\left(u\right)du=\int_{0}^{1}\left[C^{\prime }\left(u\right)-1\right]\psi \left(u\right)du \end{array}\right.$$

(using integration by parts)

(A11) $$={\left.\left[C\left(u\right)-u\right]\psi \left(u\right)\right|}_{0}^{1}-\int_{0}^{1}\left[C\left(u\right)-u\right]\psi^{\prime }\left(u\right)du$$

(using the boundness of the function $\psi \left(u\right)$, and the fact that $C\left(u\right)$ is a distribution function over the unit interval)

(A12) $$=\int_{0}^{1}\left[u-C\left(u\right)\right]\psi^{\prime }\left(u\right)du$$

(using Equation (1) and the change-of-variables $u=A\left(r\right)$)

(A13) $$=\int_{0}^{1}\left\{u-B\left[A^{-1}\left(u\right)\right]\right\}\psi^{\prime }\left(u\right)du=\int_{R}\left[A\left(r\right)-B\left(r\right)\right]\left\{\psi^{\prime }\left[A\left(r\right)\right]a\left(r\right)\right\}dr.$$

Hence, Equations (A10)–(A13) yield

(A14) $$E\left[\psi \left(U\right)\right]-E\left[\psi \left(U_{*}\right)\right]=\int_{R}\left[A\left(r\right)-B\left(r\right)\right]\left\{\psi^{\prime }\left[A\left(r\right)\right]a\left(r\right)\right\}dr.$$

Set $\psi \left(u\right)=u^{m}$, where *m* is a positive integer. The definition of the quantity $\alpha_{m}\left(Y||X\right)$ and Equation (A14) imply that

(A15) $$\left.\begin{array}{c} \alpha_{m}\left(Y||X\right)=E\left[U^{m}\right]-E\left[U_{*}^{m}\right]\\ \;\\ =\int_{R}\left[A\left(r\right)-B\left(r\right)\right]\left[mA{\left(r\right)}^{m-1}a\left(r\right)\right]dr. \end{array}\right.$$

Set $\max\left\{X_{1},\cdots ,X_{m}\right\}$ to be the maximum of *m* IID copies of the random variable *X*. The distribution function of the random variable $\max\left\{X_{1},\cdots ,X_{m}\right\}$ is

(A16) $$\left.\begin{array}{c} \mathrm{Pr}\left(\max\left\{X_{1},\cdots ,X_{m}\right\}\leq r\right)=\mathrm{Pr}\left(X_{1},\cdots ,X_{m}\leq r\right)\\ \;\\ =\mathrm{Pr}\left(X_{1}\leq r\right)\cdots \mathrm{Pr}\left(X_{m}\leq r\right)=\mathrm{Pr}{\left(X\leq r\right)}^{m}\\ \;\\ =A{\left(r\right)}^{m}. \end{array}\right.$$

Differentiating Equation (A16) implies that the density function of the random variable $\max\left\{X_{1},\cdots ,X_{m}\right\}$ is

(A17) $${\overset{ˇ}{a}}_{m}\left(r\right)=mA{\left(r\right)}^{m-1}a\left(r\right).$$

Substituting Equation (A17) into Equation (A15) yields

(A18) $$\alpha_{m}\left(Y||X\right)=\int_{R}\left[A\left(r\right)-B\left(r\right)\right]{\overset{ˇ}{a}}_{m}\left(r\right)dr.$$

Set $\psi \left(u\right)={\left(1-u\right)}^{m}$, where *m* is a positive integer. The definition of the quantity $\beta_{m}\left(Y||X\right)$ and Equation (A14) imply that

(A19) $$\left.\begin{array}{c} \beta_{m}\left(Y||X\right)=E\left[{\left(1-U_{*}\right)}^{m}\right]-E\left[{\left(1-U\right)}^{m}\right]\\ \;\\ =-\int_{R}\left[A\left(r\right)-B\left(r\right)\right]\left\{m{\left[1-A\left(r\right)\right]}^{m-1}\left[-a\left(r\right)\right]\right\}dr\\ \;\\ =\int_{R}\left[A\left(r\right)-B\left(r\right)\right]\left\{m{\left[1-A\left(r\right)\right]}^{m-1}a\left(r\right)\right\}dr. \end{array}\right.$$

Set $\min\left\{X_{1},\cdots ,X_{m}\right\}$ to be the minimum of *m* IID copies of the random variable *X*. The survival function of the random variable $\min\left\{X_{1},\cdots ,X_{m}\right\}$ is

(A20) $$\left.\begin{array}{c} \mathrm{Pr}\left(\min\left\{X_{1},\cdots ,X_{m}\right\}>r\right)=\mathrm{Pr}\left(X_{1},\cdots ,X_{m}>r\right)\\ \;\\ =\mathrm{Pr}\left(X_{1}>r\right)\cdots \mathrm{Pr}\left(X_{m}>r\right)=\mathrm{Pr}{\left(X>r\right)}^{m}\\ \;\\ ={\left[1-\mathrm{Pr}\left(X\leq r\right)\right]}^{m}={\left[1-A\left(r\right)\right]}^{m}. \end{array}\right.$$

Differentiating Equation (A20) implies that the density function of the random variable $\min\left\{X_{1},\cdots ,X_{m}\right\}$ is

(A21) $${\hat{a}}_{m}\left(r\right)=m{\left[1-A\left(r\right)\right]}^{m-1}a\left(r\right).$$

Substituting Equation (A21) into Equation (A19) yields

(A22) $$\beta_{m}\left(Y||X\right)=\int_{R}\left[A\left(r\right)-B\left(r\right)\right]{\hat{a}}_{m}\left(r\right)dr.$$

#### Appendix A.1.5. The Coincidence Likelihood **L***_m_*[*U*] and the Kullback–Leibler Limit

The density function of Equation (2), and the change in variables $u=A\left(r\right)$ imply that

(A23) $$\left.\begin{array}{c} L_{m}\left[U\right]=\int_{0}^{1}{\left[C^{\prime }\left(u\right)\right]}^{m}du\\ \;\\ =\int_{0}^{1}{\left\{\frac{b\left[A^{-1}\left(u\right)\right]}{a\left[A^{-1}\left(u\right)\right]}\right\}}^{m}du=\int_{R}{\left[\frac{b\left(r\right)}{a\left(r\right)}\right]}^{m}a\left(r\right)dr. \end{array}\right.$$

In turn, as $L_{m}\left[U_{*}\right]=1$, and as $a\left(r\right)$ is a density function, Equation (A23) implies that

(A24) $$\left.\begin{array}{c} L_{m}\left[U\right]-L_{m}\left[U_{*}\right]=\int_{R}{\left[\frac{b\left(r\right)}{a\left(r\right)}\right]}^{m}a\left(r\right)dr-1\\ \;\\ =\int_{R}{\left[\frac{b\left(r\right)}{a\left(r\right)}\right]}^{m}a\left(r\right)dr-\int_{R}a\left(r\right)dr\\ \;\\ =\int_{R}\left\{{\left[\frac{b\left(r\right)}{a\left(r\right)}\right]}^{m}-1\right\}a\left(r\right)dr. \end{array}\right.$$

In addition, Equation (A24) and L’Hopital’s rule imply that

(A25) $$\left.\begin{array}{c} \lim_{m\to 1}\frac{L_{m}\left[U\right]-L_{m}\left[U_{*}\right]}{m-1}\\ \;\\ =\lim_{m\to 1}\frac{\int_{R}\left\{{\left[\frac{b\left(r\right)}{a\left(r\right)}\right]}^{m}-1\right\}a\left(r\right)dr}{m-1}=\lim_{m\to 1}\frac{\int_{R}\left\{{\left[\frac{b\left(r\right)}{a\left(r\right)}\right]}^{m}\ln\left[\frac{b\left(r\right)}{a\left(r\right)}\right]\right\}a\left(r\right)dr}{1}\\ \;\\ =\int_{R}\left\{\left[\frac{b\left(r\right)}{a\left(r\right)}\right]\ln\left[\frac{b\left(r\right)}{a\left(r\right)}\right]\right\}a\left(r\right)dr=\int_{R}\ln\left[\frac{b\left(r\right)}{a\left(r\right)}\right]b\left(r\right)dr. \end{array}\right.$$

The quantity appearing in the bottom line of Equation (A25) is the Kullback–Leibler divergence of the random variable *Y* (whose density function is $b\left(r\right)$) from the random variable *X* (whose density function is $a\left(r\right)$).

#### Appendix A.1.6. Convexity Argument

Consider a function $\varphi \left(t\right)$ (t≥0) that is convex and that satisfies the condition $\varphi \left(1\right)=0$. Convexity implies that there is a line that coincides with the function $\varphi \left(t\right)$ at the point t=1, and that is below the the function $\varphi \left(t\right)$ at all other points t≠1. Specifically, denoting by *s* the line’s slope:

(A26) $$\varphi \left(t\right)\geq \varphi \left(1\right)+s\cdot \left(t-1\right),$$

and the inequality in Equation (A26) is strict for all t≠1. As $\varphi \left(1\right)=0$, Equation (A26) implies that

(A27) $$\varphi \left[\frac{b\left(r\right)}{a\left(r\right)}\right]\geq s\cdot \left[\frac{b\left(r\right)}{a\left(r\right)}-1\right],$$

and the inequality in Equation (A27) is strict for all *r* such that $a\left(r\right)\neq b\left(r\right)$. In turn, multiplying Equation (A27) by $a\left(r\right)$, and then integrating over the range R, implies that

(A28) $$\left.\begin{array}{c} \int_{R}\varphi \left[\frac{b\left(r\right)}{a\left(r\right)}\right]a\left(r\right)dr\geq \int_{R}\left\{s\cdot \left[\frac{b\left(r\right)}{a\left(r\right)}-1\right]\right\}a\left(r\right)dr\\ \;\\ =s\cdot \int_{R}\left[b\left(r\right)-a\left(r\right)\right]dr=s\cdot \left[\int_{R}b\left(r\right)dr-\int_{R}a\left(r\right)dr\right]\\ \;\\ =s\cdot \left(1-1\right)=0. \end{array}\right.$$

So,

(A29) $$\int_{R}\varphi \left[\frac{b\left(r\right)}{a\left(r\right)}\right]a\left(r\right)dr\geq 0,$$

and as the density function $a\left(r\right)$ is positive over the range R, the left-hand side of Equation (A29) is zero if and only if $a\left(r\right)\equiv b\left(r\right)$ (albeit for a set whose Lebesgue measure is zero). In other words, the left-hand side of Equation (A29) is zero if and only if the random variables *X* and *Y* are equal in law.

### Appendix A.2. Derivations: Section 3

#### Appendix A.2.1. Derivation of Equation (15)

In what follows, $F\left(w\right)=\mathrm{Pr}\left(W\leq w\right)$ (0<w<∞) is the distribution function of the random variable *W*, and $f_{\$}\left(w\right)=\frac{1}{\mu }wf\left(w\right)$ (0<w<∞) is the density function of the random variable $W_{\$}$. Setting X=W and $Y=W_{\$}$ (the equalities being in law) implies that $A\left(r\right)=F\left(r\right)$ and $b\left(r\right)=f_{\$}\left(r\right)$, where $R=\left(0,\infty \right)$. In turn, Equation (A5) implies that

(A30) $$\left.\begin{array}{c} \mathrm{Pr}\left(X_{1},\cdots ,X_{m}\leq Y\right)=\int_{R}A{\left(r\right)}^{m}b\left(r\right)dr\\ \;\\ =\int_{0}^{\infty }F{\left(w\right)}^{m}f_{\$}\left(w\right)dw=\int_{0}^{\infty }F{\left(w\right)}^{m}\left[\frac{1}{\mu }wf\left(w\right)\right]dw\\ \;\\ =\frac{1}{\left(m+1\right)\mu }\int_{0}^{\infty }w\left[\left(m+1\right)F{\left(w\right)}^{m}f\left(w\right)\right]dw. \end{array}\right.$$

Set $\vee_{m+1}=\max\left\{W_{1},\cdots ,W_{m+1}\right\}$ to be the maximum of m+1 IID copies of the random variable *W*. The distribution function of the random variable $\vee_{m+1}$ is

(A31) $$\left.\begin{array}{c} \mathrm{Pr}\left(\vee_{m+1}\leq w\right)=\mathrm{Pr}\left(W_{1},\cdots ,W_{m+1}\leq w\right)\\ \;\\ =\mathrm{Pr}\left(W_{1}\leq w\right)\cdots \mathrm{Pr}\left(W_{m+1}\leq w\right)=F{\left(w\right)}^{m+1}. \end{array}\right.$$

Differentiating Equation (A31) implies that the density function of the random variable $\vee_{m+1}$ is

(A32) $$\frac{d}{dw}\mathrm{Pr}\left(\vee_{m+1}\leq w\right)=\left(m+1\right)F{\left(w\right)}^{m}f\left(w\right).$$

In turn, Equation (A32) implies that the mean of the random variable $\vee_{m+1}$ is

(A33) $$E\left[\vee_{m+1}\right]=\int_{0}^{\infty }w\left[\left(m+1\right)F{\left(w\right)}^{m}f\left(w\right)\right]dw.$$

Combined together, Equations (A30) and (A33) imply that

(A34) $$\mathrm{Pr}\left(X_{1},\cdots ,X_{m}\leq Y\right)=\frac{E\left[\vee_{m+1}\right]}{\left(m+1\right)\mu }.$$

Substituting Equation (A34) into Equation (4) yields

(A35) $$\left.\begin{array}{c} \alpha_{m}\left(Y||X\right)=\left(m+1\right)\mathrm{Pr}\left(X_{1},\cdots ,X_{m}\leq Y\right)-1\\ \;\\ =\frac{E\left[\vee_{m+1}\right]}{\mu }-1=\frac{E\left[\vee_{m+1}\right]-\mu }{\mu }, \end{array}\right.$$

and thus we obtain that

(A36) $$\alpha_{m}\left(W_{\$}\left|\right|W\right)=\frac{E\left[\max\left\{W_{1},\cdots ,W_{m+1}\right\}\right]-E\left[W\right]}{E\left[W\right]}.$$

Note that $W_{1}\leq \max\left\{W_{1},\cdots ,W_{m+1}\right\}\leq W_{1}+\cdots +W_{m+1}$, and hence

(A37) $$\left.\begin{array}{c} E\left[W\right]=E\left[W_{1}\right]\leq E\left[\max\left\{W_{1},\cdots ,W_{m+1}\right\}\right]\\ \;\\ \leq E\left[W_{1}+\cdots +W_{m+1}\right]=E\left[W_{1}\right]+\cdots +E\left[W_{m+1}\right]\\ \;\\ =\left(m+1\right)E\left[W\right]. \end{array}\right.$$

In turn, Equation (A37) implies that

(A38) $$0\leq E\left[\max\left\{W_{1},\cdots ,W_{m+1}\right\}\right]-E\left[W\right]\leq mE\left[W\right].$$

Combined together, Equations (A36) and (A38) imply that

(A39) $$0\leq \alpha_{m}\left(W_{\$}\left|\right|W\right)\leq m.$$

#### Appendix A.2.2. Derivation of Equation (16)

In what follows, $\bar{F}\left(w\right)=\mathrm{Pr}\left(W>w\right)$ (0<w<∞) is the survival function of the random variable *W*, and $f_{\$}\left(w\right)=\frac{1}{\mu }wf\left(w\right)$ (0<w<∞) is the density function of the random variable $W_{\$}$. Setting X=W and $Y=W_{\$}$ (the equalities being in law) implies that $\bar{A}\left(r\right)=\bar{F}\left(r\right)$ and $b\left(r\right)=f_{\$}\left(r\right)$, where $R=\left(0,\infty \right)$. In turn, Equation (A8) implies that

(A40) $$\left.\begin{array}{c} \mathrm{Pr}\left(X_{1},\cdots ,X_{m}>Y\right)=\int_{R}\bar{A}{\left(r\right)}^{m}b\left(r\right)dr\\ \;\\ =\int_{0}^{\infty }\bar{F}{\left(w\right)}^{m}f_{\$}\left(w\right)dw=\int_{0}^{\infty }\bar{F}{\left(w\right)}^{m}\left[\frac{1}{\mu }wf\left(w\right)\right]dw\\ \;\\ =\frac{1}{\left(m+1\right)\mu }\int_{0}^{\infty }w\left[\left(m+1\right)\bar{F}{\left(w\right)}^{m}f\left(w\right)\right]dw. \end{array}\right.$$

Set $\wedge_{m+1}=\min\left\{W_{1},\cdots ,W_{m+1}\right\}$ to be the minimum of m+1 IID copies of the random variable *W*. The survival function of the random variable $\wedge_{m+1}$ is

(A41) $$\left.\begin{array}{c} \mathrm{Pr}\left(\wedge_{m+1}>w\right)=\mathrm{Pr}\left(W_{1},\cdots ,W_{m+1}>w\right)\\ \;\\ =\mathrm{Pr}\left(W_{1}>w\right)\cdots \mathrm{Pr}\left(W_{m+1}>w\right)=\bar{F}{\left(w\right)}^{m+1}. \end{array}\right.$$

Differentiating Equation (A41) implies that the density function of the random variable $\wedge_{m+1}$ is

(A42) $$-\frac{d}{dw}\mathrm{Pr}\left(\wedge_{m+1}>w\right)=\left(m+1\right)\bar{F}{\left(w\right)}^{m}f\left(w\right).$$

In turn, Equation (A42) implies that the mean of the random variable $\wedge_{m+1}$ is

(A43) $$E\left[\wedge_{m+1}\right]=\int_{0}^{\infty }w\left[\left(m+1\right)\bar{F}{\left(w\right)}^{m}f\left(w\right)\right]dw.$$

Combined together, Equations (A40) and (A43) imply that

(A44) $$\mathrm{Pr}\left(X_{1},\cdots ,X_{m}>Y\right)=\frac{E\left[\wedge_{m+1}\right]}{\left(m+1\right)\mu }.$$

Substituting Equation (A44) into Equation (5) yields

(A45) $$\left.\begin{array}{c} \beta_{m}\left(Y||X\right)=1-\left(m+1\right)\mathrm{Pr}\left(X_{1},\cdots ,X_{m}>Y\right)\\ \;\\ =1-\frac{E\left[\wedge_{m+1}\right]}{\mu }=\frac{\mu -E\left[\wedge_{m+1}\right]}{\mu }, \end{array}\right.$$

and thus we obtain that

(A46) $$\beta_{m}\left(W_{\$}\left|\right|W\right)=\frac{E\left[W\right]-E\left[\min\left\{W_{1},\cdots ,W_{m+1}\right\}\right]}{E\left[W\right]}.$$

Note that $0\leq \min\left\{W_{1},\cdots ,W_{m+1}\right\}\leq W_{1}$, and hence

(A47) $$\left.\begin{array}{c} 0\leq E\left[\min\left\{W_{1},\cdots ,W_{m+1}\right\}\right]\\ \;\\ \leq E\left[W_{1}\right]=E\left[W\right] \end{array}\right.$$

In turn, Equation (A37) implies that

(A48) $$0\leq E\left[W\right]-E\left[\min\left\{W_{1},\cdots ,W_{m+1}\right\}\right]\leq E\left[W\right].$$

Combined together, Equations (A46) and (A48) imply that

(A49) $$0\leq \beta_{m}\left(W_{\$}\left|\right|W\right)\leq 1.$$

#### Appendix A.2.3. Derivation of Equations (19) and (20)

In what follows, $f\left(w\right)$ (0<w<∞) is the density function of the random variable *W*, and $f_{\$}\left(w\right)=\frac{1}{\mu }wf\left(w\right)$ (0<w<∞) is the density function of the random variable $W_{\$}$. As noted above, the ratio of the density function of $W_{\$}$ to the density function of *W* is $f_{\$}\left(w\right)/f\left(w\right)=w/\mu$.

Setting X=W and $Y=W_{\$}$ (the equalities being in law) implies that $a\left(r\right)=f\left(r\right)$ and $b\left(r\right)=f_{\$}\left(r\right)$, where $R=\left(0,\infty \right)$. In turn, Equation (10) implies that

(A50) $$\left.\begin{array}{c} \gamma_{m}\left(Y||X\right)=\int_{R}\left\{{\left[\frac{b\left(r\right)}{a\left(r\right)}\right]}^{m}-1\right\}a\left(r\right)dr\\ \;\\ =\int_{0}^{\infty }\left\{{\left[\frac{f_{\$}\left(w\right)}{f\left(w\right)}\right]}^{m}-1\right\}f\left(w\right)dw=\int_{0}^{\infty }\left[{\left(\frac{w}{\mu }\right)}^{m}-1\right]f\left(w\right)dw\\ \;\\ =\frac{1}{\mu^{m}}\int_{0}^{\infty }w^{m}f\left(w\right)dw-\int_{0}^{\infty }f\left(w\right)dw\\ \;\\ =\frac{E\left[W^{m}\right]}{E{\left[W\right]}^{m}}-1, \end{array}\right.$$

and thus we obtain that

(A51) $$\gamma_{m}\left(W_{\$}\left|\right|W\right)=\frac{E\left[W^{m}\right]-E{\left[W\right]}^{m}}{E{\left[W\right]}^{m}}.$$

Setting $X=W_{\$}$ and Y=W (the equalities being in law) implies that $a\left(r\right)=f_{\$}\left(r\right)$ and $b\left(w\right)=f\left(r\right)$, where $R=\left(0,\infty \right)$. In turn, Equation (10) implies that

(A52) $$\left.\begin{array}{c} \gamma_{m}\left(Y||X\right)=\int_{R}\left\{{\left[\frac{b\left(r\right)}{a\left(r\right)}\right]}^{m}-1\right\}a\left(r\right)dr\\ \;\\ =\int_{0}^{\infty }\left\{{\left[\frac{f\left(w\right)}{f_{\$}\left(w\right)}\right]}^{m}-1\right\}f_{\$}\left(w\right)dw=\int_{0}^{\infty }\left[{\left(\frac{\mu }{w}\right)}^{m}-1\right]\left[\frac{1}{\mu }wf\left(w\right)\right]dw\\ \;\\ =\mu^{m-1}\int_{0}^{\infty }w^{1-m}f\left(w\right)dw-\frac{1}{\mu }\int_{0}^{\infty }wf\left(w\right)dw\\ \;\\ =\frac{E\left[W^{1-m}\right]}{E{\left[W\right]}^{1-m}}-1, \end{array}\right.$$

and thus we obtain that

(A53) $$\gamma_{m}\left(W||W_{\$}\right)=\frac{E\left[W^{1-m}\right]-E{\left[W\right]}^{1-m}}{E{\left[W\right]}^{1-m}}.$$

Consider a smooth function $\varphi \left(t\right)$ that is defined over the positive half-line (t>0), and that is convex: $\varphi^{\prime \prime }\left(t\right)>0$. Jensen’s inequality [59] asserts that

(A54) $$\varphi \left(E\left[W\right]\right)\leq E\left[\varphi \left(W\right)\right].$$

Jensen’s inequality further asserts that an equality sign holds in Equation (A54) if and only if the random variable *W* is constant with probability one: $\varphi \left(E\left[W\right]\right)=E\left[\varphi \left(W\right)\right]\Leftrightarrow \mathrm{Pr}\left(W=c\right)$, where *c* is a positive constant. For m>1 the function $\varphi \left(t\right)=t^{m}$ is convex, and hence Equation (A54) implies that $E{\left[W\right]}^{m}\leq E\left[W^{m}\right]$; thus Equation (A51) implies that $\gamma_{m}\left(W_{\$}\left|\right|W\right)\geq 0$. For m>1 the function $\varphi \left(t\right)=t^{1-m}$ is convex, and hence Equation (A54) implies that $E{\left[W\right]}^{1-m}\leq E\left[W^{1-m}\right]$; thus Equation (A53) implies that $\gamma_{m}\left(W||W_{\$}\right)\geq 0$. Moreover, the quantities $\gamma_{m}\left(W_{\$}\left|\right|W\right)$ and $\gamma_{m}\left(W_{\$}\left|\right|W\right)$ are zero if and only if the random variable *W* is constant with probability one.

### Appendix A.3. Derivations: Section 4

#### Appendix A.3.1. Derivation of Equation (24)

In what follows, $F\left(t\right)=\mathrm{Pr}\left(T\leq t\right)$ (0<t<∞) is the distribution function of the random variable *T*, and $\bar{F}\left(t\right)=\mathrm{Pr}\left(T>t\right)=1-F\left(t\right)$ (0<t<∞) is the corresponding survival function. In addition, $f_{res}\left(t\right)=\frac{1}{\mu }\bar{F}\left(t\right)$ (0<t<∞) is the density function of the random variable $T_{res}$. Setting X=T and $Y=T_{res}$ (the equalities being in law) implies that $A\left(r\right)=F\left(r\right)$ and $b\left(r\right)=f_{res}\left(r\right)$, where $R=\left(0,\infty \right)$. In turn, Equation (A5) implies that

(A55) $$\left.\begin{array}{c} \mathrm{Pr}\left(X_{1},\cdots ,X_{m}\leq Y\right)=\int_{R}A{\left(r\right)}^{m}b\left(r\right)dr\\ \;\\ =\int_{0}^{\infty }F{\left(t\right)}^{m}f_{res}\left(t\right)dt=\int_{0}^{\infty }F{\left(t\right)}^{m}\left[\frac{1}{\mu }\bar{F}\left(t\right)\right]dt\\ \;\\ =\frac{1}{\mu }\int_{0}^{\infty }F{\left(t\right)}^{m}\left[1-F\left(t\right)\right]dt=\frac{1}{\mu }\int_{0}^{\infty }\left[F{\left(t\right)}^{m}-F{\left(t\right)}^{m+1}\right]dt\\ \;\\ =\frac{1}{\mu }\int_{0}^{\infty }\left\{\left[1-F{\left(t\right)}^{m+1}\right]-\left[1-F{\left(t\right)}^{m}\right]\right\}dt\\ \;\\ =\frac{1}{\mu }\left\{\int_{0}^{\infty }\left[1-F{\left(t\right)}^{m+1}\right]dt-\int_{0}^{\infty }\left[1-F{\left(t\right)}^{m}\right]dt\right\}. \end{array}\right.$$

Set $\vee_{k}=\max\left\{T_{1},\cdots ,T_{k}\right\}$ to be the maximum of *k* IID copies of the random variable *T*. The distribution function of the random variable $\vee_{k}$ is

(A56) $$\left.\begin{array}{c} \mathrm{Pr}\left(\vee_{k}\leq t\right)=\mathrm{Pr}\left(T_{1},\cdots ,T_{k}\leq t\right)\\ \;\\ =\mathrm{Pr}\left(T_{1}\leq t\right)\cdots \mathrm{Pr}\left(T_{k}\leq t\right)=F{\left(t\right)}^{k}. \end{array}\right.$$

In turn, using Equation (A56), the mean of the random variable $\vee_{k}$ is

(A57) $$\left.\begin{array}{c} E\left[\vee_{k}\right]=\int_{0}^{\infty }\mathrm{Pr}\left(\vee_{k}>t\right)dt\\ \;\\ =\int_{0}^{\infty }\left[1-\mathrm{Pr}\left(\vee_{k}\leq t\right)\right]dt=\int_{0}^{\infty }\left[1-F{\left(t\right)}^{k}\right]dt. \end{array}\right.$$

Combined together, Equations (A55) and (A57) imply that

(A58) $$\mathrm{Pr}\left(X_{1},\cdots ,X_{m}\leq Y\right)=\frac{1}{\mu }\left\{E\left[\vee_{m+1}\right]-E\left[\vee_{m}\right]\right\},$$

where $\left\{T_{1},\cdots ,T_{m+1}\right\}$ are m+1 IID copies of the random variable *T*; and $\vee_{m}=\max\left\{T_{1},\cdots ,T_{m}\right\}$, and $\vee_{m+1}=\max\left\{T_{1},\cdots ,T_{m+1}\right\}$.

Substituting Equation (A58) into Equation (4) yields

(A59) $$\left.\begin{array}{c} \alpha_{m}\left(Y||X\right)=\left(m+1\right)\mathrm{Pr}\left(X_{1},\cdots ,X_{m}\leq Y\right)-1\\ \;\\ =\left(m+1\right)\frac{1}{\mu }\left\{E\left[\vee_{m+1}\right]-E\left[\vee_{m}\right]\right\}-1, \end{array}\right.$$

and thus we obtain that

(A60) $$\alpha_{m}\left(T_{res}\left|\right|T\right)=\left(m+1\right)\frac{E\left[\max\left\{T_{1},\cdots ,T_{m+1}\right\}\right]-E\left[\max\left\{T_{1},\cdots ,T_{m}\right\}\right]}{E\left[T\right]}-1.$$

#### Appendix A.3.2. Derivation of Equation (25)

In what follows, $\bar{F}\left(t\right)=\mathrm{Pr}\left(T>t\right)$ (0<t<∞) is the survival function of the random variable *T*, and $f_{res}\left(t\right)=\frac{1}{\mu }\bar{F}\left(t\right)$ (0<t<∞) is the density function of the random variable $T_{res}$. Setting X=T and $Y=T_{res}$ (the equalities being in law) implies that $\bar{A}\left(r\right)=\bar{F}\left(r\right)$ and $b\left(r\right)=f_{res}\left(r\right)$, where $R=\left(0,\infty \right)$. In turn, Equation (A8) implies that

(A61) $$\left.\begin{array}{c} \mathrm{Pr}\left(X_{1},\cdots ,X_{m}>Y\right)=\int_{R}\bar{A}{\left(r\right)}^{m}b\left(r\right)dr\\ \;\\ =\int_{0}^{\infty }\bar{F}{\left(t\right)}^{m}f_{res}\left(t\right)dt=\int_{0}^{\infty }\bar{F}{\left(t\right)}^{m}\left[\frac{1}{\mu }\bar{F}\left(t\right)\right]dt\\ \;\\ =\frac{1}{\mu }\int_{0}^{\infty }\bar{F}{\left(t\right)}^{m+1}dt. \end{array}\right.$$

Set $\wedge_{m+1}=\min\left\{T_{1},\cdots ,T_{m+1}\right\}$ to be the minimum of m+1 IID copies of the random variable *T*. The survival function of the random variable $\wedge_{m+1}$ is

(A62) $$\left.\begin{array}{c} \mathrm{Pr}\left(\wedge_{m+1}>t\right)=\mathrm{Pr}\left(T_{1},\cdots ,T_{m+1}>t\right)\\ \;\\ =\mathrm{Pr}\left(T_{1}>t\right)\cdots \mathrm{Pr}\left(T_{m+1}>t\right)=\bar{F}{\left(t\right)}^{m+1}. \end{array}\right.$$

In turn, Equation (A32) implies that the mean of the random variable $\wedge_{m+1}$ is

(A63) $$E\left[\wedge_{m+1}\right]=\int_{0}^{\infty }\mathrm{Pr}\left(\wedge_{m+1}>t\right)dt=\int_{0}^{\infty }\bar{F}{\left(t\right)}^{m+1}dt.$$

Substituting Equation (A63) into Equation (A61) implies that

(A64) $$\mathrm{Pr}\left(X_{1},\cdots ,X_{m}>Y\right)=\frac{1}{\mu }E\left[\wedge_{m+1}\right].$$

In turn, substituting Equation (A64) into Equation (5) yields

(A65) $$\left.\begin{array}{c} \beta_{m}\left(Y||X\right)=1-\left(m+1\right)\mathrm{Pr}\left(X_{1},\cdots ,X_{m}>Y\right)\\ \;\\ =1-\left(m+1\right)\frac{1}{\mu }E\left[\wedge_{m+1}\right], \end{array}\right.$$

and thus we obtain that

(A66) $$\beta_{m}\left(T_{res}\left|\right|T\right)=1-\left(m+1\right)\frac{E\left[\min\left\{T_{1},\cdots ,T_{m+1}\right\}\right]}{E\left[T\right]}.$$

#### Appendix A.3.3. The Quantities *γ_m_*(*T_res_*||*T*) and *γ_m_*(*T*||*T_res_*)

In what follows, $f\left(t\right)$ (0<t<∞) is the density function of the random variable *T*, and $f_{res}\left(t\right)=\frac{1}{\mu }\bar{F}\left(t\right)$ (0<t<∞) is the density function of the random variable $T_{res}$. As noted above, the ratio of the density function of $T_{res}$ to the density function of *T* is $f_{res}\left(t\right)/f\left(t\right)=1/\left[\mu h\left(t\right)\right]$, where $h\left(t\right)$ is the hazard rate of the random variable *T*.

Setting X=T and $Y=T_{res}$ (the equalities being in law) implies that $a\left(r\right)=f\left(r\right)$ and $b\left(r\right)=f_{res}\left(r\right)$, where $R=\left(0,\infty \right)$. In turn, Equation (10) implies that

(A67) $$\left.\begin{array}{c} \gamma_{m}\left(T_{res}\left|\right|T\right)=\int_{R}\left\{{\left[\frac{b\left(r\right)}{a\left(r\right)}\right]}^{m}-1\right\}a\left(r\right)dr\\ \;\\ =\int_{0}^{\infty }\left\{{\left[\frac{f_{res}\left(t\right)}{f\left(t\right)}\right]}^{m}-1\right\}f\left(t\right)dt=\int_{0}^{\infty }\left\{{\left[\frac{1}{\mu h\left(t\right)}\right]}^{m}-1\right\}f\left(t\right)dt\\ \;\\ =\mu^{-m}\int_{0}^{\infty }h{\left(t\right)}^{-m}f\left(t\right)dt-\int_{0}^{\infty }f\left(t\right)dt\\ \;\\ =E{\left[T\right]}^{-m}E\left[h{\left(T\right)}^{-m}\right]-1. \end{array}\right.$$

Setting $X=T_{res}$ and Y=T (the equalities being in law) implies that $a\left(r\right)=f_{res}\left(r\right)$ and $b\left(r\right)=f\left(r\right)$, where $R=\left(0,\infty \right)$. In turn, Equation (10) implies that

(A68) $$\left.\begin{array}{c} \gamma_{m}\left(T||T_{res}\right)=\int_{R}\left\{{\left[\frac{b\left(r\right)}{a\left(r\right)}\right]}^{m}-1\right\}a\left(r\right)dr\\ \;\\ =\int_{0}^{\infty }\left\{{\left[\frac{f\left(t\right)}{f_{res}\left(t\right)}\right]}^{m}-1\right\}f_{res}\left(t\right)dt=\int_{0}^{\infty }\left\{{\left[\mu h\left(t\right)\right]}^{m}-1\right\}f_{res}\left(t\right)dt\\ \;\\ =\mu^{m}\int_{0}^{\infty }h{\left(t\right)}^{m}f_{res}\left(t\right)dt-\int_{0}^{\infty }f_{res}\left(t\right)dt\\ \;\\ =E{\left[T\right]}^{m}E\left[h{\left(T_{res}\right)}^{m}\right]-1. \end{array}\right.$$

#### Appendix A.3.4. Estimation of the Curve *C*(*u*) for the Renewal Application

In the opening of Section 4, we described how the random variable $T_{res}$—the “residual lifetime” of the random variable *T*—is constructed via a renewal process that is generated from the random variable *T*. We now describe an alternative construction of the random variable $T_{res}$; this construction will also be based on a renewal process that is generated from the random variable *T*. In what follows, $\left\{T_{1},T_{2},\cdots ,T_{n}\right\}$ are IID copies of the random variable *T*.

The first *n* renewal epochs of the renewal process are the temporal points $\tau_{i}=T_{1}+\cdots +T_{i}$ (i=1,⋯,n). Now, sample at random a time point over the interval $\left[0,\tau_{n}\right]$, and set $D_{n}$ to be the distance from the time point (which was sampled at random) to the first renewal epoch after it. In other words, $D_{n}$ is the distance from the time point (which was sampled at random) to nearest renewal epoch to its right (on the temporal time axis). The distance $D_{n}$ converges in law, in the limit n→∞, to the random variable $T_{res}$.

Given *n* samples of the random variable *T*, order these samples increasingly: $t_{1}\leq t_{2}\leq \cdots \leq t_{n-1}\leq t_{n}$. Based on these samples, set $A^{-1}\left(u\right)$ to be the inverse of the empirical distribution function of the random variable *T*. This inverse function passes through the following points:

(A69) $$A^{-1}\left(\frac{i}{n}\right)=t_{i}$$

(i=1,⋯,n).

Based on the above samples, set $B\left(t\right)$ to be the empirical distribution function of the random variable $D_{n}$. This distribution function is given by

(A70) $$B\left(t\right)=\frac{\min\left\{t,t_{1}\right\}+\min\left\{t,t_{2}\right\}+\cdots +\min\left\{t,t_{n}\right\}}{t_{1}+t_{2}+\cdots +t_{n}}.$$

In particular, note that

(A71) $$\left.\begin{array}{c} B\left(t_{i}\right)=\frac{t_{1}+\cdots +t_{i-1}+\left(n-i+1\right)t_{i}}{t_{1}+t_{2}+\cdots +t_{n}}\\ \;\\ =\frac{t_{1}+\cdots +t_{i}+\left(n-i\right)t_{i}}{t_{1}+t_{2}+\cdots +t_{n}}\\ \;\\ =\left(\pi_{1}+\cdots +\pi_{i}\right)+\left(n-i\right)\pi_{i}, \end{array}\right.$$

where $\pi_{i}=t_{i}/\left(t_{1}+\cdots +t_{n}\right)$ (i=1,2,⋯,n).

Equations (1) and (A69) imply that

(A72) $$C\left(\frac{i}{n}\right)=B\left[A^{-1}\left(\frac{i}{n}\right)\right]=B\left(t_{i}\right),$$

and hence Equation (A71) yields

(A73) $$C\left(\frac{i}{n}\right)=\left(\pi_{1}+\cdots +\pi_{i}\right)+\left(n-i\right)\pi_{i}.$$

In turn, from Equation (A73) it follows that

(A74) $$\frac{C\left(\frac{i}{n}\right)-C\left(\frac{i-1}{n}\right)}{\frac{1}{n}}=n\left(n-i+1\right)\left(\pi_{i}-\pi_{i-1}\right),$$

where $\pi_{0}=0$.
